# Multi‐trait/environment sparse genomic prediction using the SFSI R‐package

**DOI:** 10.1002/tpg2.70050

**Published:** 2025-06-13

**Authors:** Marco Lopez‐Cruz, Gustavo de los Campos

**Affiliations:** ^1^ Department of Epidemiology and Biostatistics Michigan State University East Lansing Michigan USA; ^2^ Institute for Quantitative Health Science and Engineering Michigan State University East Lansing Michigan USA; ^3^ Department of Statistics and Probability Michigan State University East Lansing Michigan USA

## Abstract

Sparse selection indices (SSIs) can be used to predict the genetic merit of selection candidates using high‐dimensional phenotypes (e.g., crop imaging) measured on each of the candidates of selection. Unlike traditional selection indices, SSIs can perform variable selection, thus enabling borrowing of information from a subset of the measured phenotypes. Likewise, sparse genomic prediction (SGP) can be used to predict genetic merit by borrowing information from a subset of the training dataset. In this study, we introduce a framework for multi‐trait/environment SGP (MT‐SGP) that combines the features of SSI and SGP into a single model. For candidates of selection, an MT‐SGP produces prediction equations that use subsets of the training data, borrowing information from correlated traits expressed in training genotypes that are genetically close to the candidates of selection. Along with the methodology, we present an R‐package (sparse family and selection index) that provides functions to solve SSIs, SGP, and MT‐SGP problems. After presenting simplified examples that illustrate the use of the functions included in the package, we provide extensive benchmarks (using three data sets covering three crops and 30 traits/environments). Our results suggest that MT‐SGP either outperforms (with up to 15% gains in prediction accuracy) or performs similarly to MT‐genomic best linear unbiased prediction. The benchmarks provide insight regarding the conditions (sample size, genetic correlation among traits, and trait heritability) under which the use of MT‐SGP can lead to gains in prediction accuracy.

## Core Ideas

- We present a framework for multi‐trait‐sparse genomic prediction (MT‐SGP) that combines the features of SSI and SGP into a unified setting.
- Along with the methodology, we present an R‐package that can be used to solve SSI, SGP, and MT‐SGP problems.
- The MT‐SGP produces equations that select training phenotypes and individuals from where the predictions are derived.
- The relative superiority of the MT‐SGP over the multi‐trait genomic best linear unbiased prediction (GBLUP) (MT‐GBLUP) is largely influenced by the sample size, trait heritability, and genetic correlations.

**Abbreviations**
GBLUP: genomic best linear unbiased prediction
GRM: genomic relationship matrix
LARS: least angle regression
LASSO: least absolute shrinkage and selection operator
MSPE: mean squared prediction error
MT: multi‐trait
MT‐SGP: multi‐trait sparse genomic prediction
RSS: residual sum of squares
SFSI: sparse family and selection index
SGP: sparse genomic prediction
SI: selection index
SNP: single‐nucleotide polymorphism
SSI: sparse selection index
ST: single‐trait
TRN: training
TST: testing

## 1 INTRODUCTION

The increasing availability of high‐dimensional genotype and phenotype data have created new opportunities to improve breeding‐value prediction and selection accuracy (Cabrera‐Bosquet et al., 2012; Cobb et al., 2013). However, high‐dimensional genomic models are prone to overfitting. To avoid this, model parameters (e.g., single‐nucleotide polymorphism [SNP] effects) are often estimated using penalized regression methods such as Ridge‐Regression (Hoerl & Kennard, 1970), least absolute shrinkage and selection operator (LASSO) (Tibshirani, 1996), and Elastic‐Net (Zou & Hastie, 2005).

Genomic prediction methods were originally developed for single‐trait/environment prediction problems (e.g., Meuwissen et al., 2001); soon after, these methods were extended to multi‐trait/multi‐environment models involving a few phenotypes/environments (e.g., Burgueño et al., 2012). Modern high‐throughput phenotyping technologies can produce information on hundreds or potentially thousands of phenotypes. One way to use these phenotypes for breeding decisions is through the use of selection indices (SIs; Hazel, 1943; Smith, 1936). However, the SI methodology was originally developed for cases involving a few measured phenotypes. As with other high‐dimensional regression problems, SIs using high‐dimensional phenotypes (e.g., crop imaging) are prone to overfitting, which leads to an SI with low heritability and, hence, low accuracy of indirect selection (Lopez‐Cruz et al., 2020). To avoid these problems, Lopez‐Cruz et al. (2020) developed sparse selection indices (SSIs) that integrate the standard SI methodology with sparsity‐inducing techniques used in penalized regressions.

Interestingly, the same equations of the SSI can be used for sparse genomic prediction (SGP, Lopez‐Cruz & de los Campos, 2021). An SGP model derives predictions for candidates of selection using a subset of the training dataset. The SSI method derives breeding value prediction by borrowing information from correlated traits measured within an individual, and the SGP method predicts breeding values for a phenotype by borrowing information from measurements of the same phenotype on genetically related individuals. Naturally, these two methodologies can be combined into a framework to derive predictions that borrow information within‐individual‐between‐traits and between‐individuals‐within‐trait. Therefore, in this study, we integrate the SSI and SGP approaches into a unified framework for multi‐trait/environment sparse genomic prediction (MT‐SGP). The MT‐SGP model can be used to derive predictions for the genetic merit of candidates of selection for several selection objectives using multi‐trait/environment genomic data.

It turns out that the BLUPs of genetic values from mixed‐effect models and the standard SI are mathematically equivalent (Henderson, 1963); thus, the genomic BLUP (genomic best linear unbiased prediction [GBLUP]; VanRaden, 2007, 2008) appears to be a particular instance of the SGP. Likewise, the multi‐trait/environment GBLUP (MT‐GBLUP) happens to be a special case of the MT‐SGP. However, unlike the MT‐GBLUP (which produces prediction equations that borrow information from all the available training data), an MT‐SGP derives predictions using an optimal subset of training data, borrowing information from genetically correlated traits observed in individuals that are genetically close to the candidates of selection. Along with the methodology, we introduce the sparse family and selection index (SFSI) R‐package, which offers functionality to solve SSI, SGP, and MT‐SGP problems.

After introducing the MT‐SGP model, we offer a few simplified examples illustrating the use of the SFSI R‐package. Subsequently, we present extensive benchmarks comparing MT‐SGP with the MT‐GBLUP counterpart, using three crop data sets (wheat, *Triticum aestivum*; maize, *Zea mays*; and rice, *Oryza sativa*) covering 30 traits/environments. Finally, we provide results from a computational benchmark and discuss the conditions (sample size, genetic correlations among traits, and trait heritabilities) for which MT‐SGP is likely to deliver gains in prediction accuracy (PA) relative to MT‐GBLUP.

## 2 MATERIALS AND METHODS

We begin this section by providing an overview of penalized regressions, SSI, and single‐trait SGP (ST‐SGP) problems. Subsequently, we introduce the MT‐SGP, which includes SSI and ST‐SGP as special cases. As we review these prediction methods, we provide simplified examples of how functions included in the SFSI R‐package can be used to solve each of these problems.

### 2.1 Penalized regression using sufficient statistics

In a linear regression model, an n‐vector outcome, $y={\left(y_{1},\text{…},y_{n}\right)}^{\prime }$, is regressed on p predictors, $X=\{x_{ij}\}$ (an n×p matrix), using a model of the form y=Xβ+ε, where $\beta ={\left(\beta_{1},\text{…},\beta_{p}\right)}^{\prime }$ is a p‐vector of regression coefficients and $\varepsilon ={\left(\varepsilon_{1},\text{…},\varepsilon_{n}\right)}^{\prime }$ is an n‐vector of random error terms.

In a penalized linear regression, the regression coefficients are estimated by minimizing an objective function that is the sum of the residual sum of squares, $\mathrm{RSS}={(y\;-\;X\beta }^{)\;}\left(y\;-\;X\beta \right)$ and a penalty on model complexity. Common choices for the penalty function include the L1‐norm: $\sum_{j=1}^{p}\left|\beta_{j}\right|$ (LASSO; Tibshirani, 1996), the L2‐norm: $\sum_{j=1}^{p}\beta_{j}^{2}$ (Ridge Regression; Hoerl & Kennard, 1970), or a linear combination of the two (Elastic‐Net; Zou & Hastie, 2005). The Elastic‐Net penalized RSS problem can be expressed as follows:

$$\hat{\beta }=\underset{\beta }{arg\;\min}\;\left[\frac{1}{2n}{\left(y\;-\;X\beta \right)}^{\prime }\left(y\;-\;X\beta \right)+\lambda F\left(\beta \right)\right]$$

where λ is a regularization (or penalty) parameter and $F\left(\beta \right)=\alpha \sum_{j=1}^{p}\left|\beta_{j}\right|+\frac{1}{2}\left(1\;-\alpha \right)\sum_{j=1}^{p}\beta_{j}^{2}$ is a penalty function on β with α∈[0,1] being a numeric value controlling the weights of the L1 and L2 penalty norms. The RSS ${\left(y\;-\;X\beta \right)}^{\prime }\left(y\;-\;X\beta \right)$ in the right‐hand side can be expanded as $y^{\prime }y+\beta^{\prime }X^{\prime }X\beta -\;2\beta^{\prime }X^{\prime }y$. The term $y^{\prime }y$ does not involve the unknown coefficients β, thus, it can be removed from the objective function without altering the solution. Therefore, the above objective function can be expressed as follows:

(1) $$\hat{\beta }=\underset{\beta }{arg\;\min}\;\left[\frac{1}{2}\beta^{\prime }\Sigma \beta -\beta^{\prime }\Gamma +\lambda F\left(\beta \right)\right]$$

where $\Sigma =\frac{1}{n}\;X^{\prime }X$ and $\Gamma =\frac{1}{n}\;X^{\prime }y$ are the sufficient statistics for linear models that correspond (if the outcome and predictors are centered to a zero mean) to the method‐of‐moments estimates of the (co)variance matrix of the predictors, and the covariance matrix between predictors and the response, respectively.

**Core Ideas**

- We present a framework for multi‐trait‐sparse genomic prediction (MT‐SGP) that combines the features of SSI and SGP into a unified setting.
- Along with the methodology, we present an R‐package that can be used to solve SSI, SGP, and MT‐SGP problems.
- The MT‐SGP produces equations that select training phenotypes and individuals from where the predictions are derived.
- The relative superiority of the MT‐SGP over the multi‐trait genomic best linear unbiased prediction (GBLUP) (MT‐GBLUP) is largely influenced by the sample size, trait heritability, and genetic correlations.

In the SFSI R‐package, the functions LARS() and solveEN() can be used to obtain solutions to the optimization problem in Equation (1) taking Σ and Γ as inputs. The function LARS() provides LASSO solutions for the entire path $\left\{\lambda_{\max}=\lambda_{1}>\lambda_{2}>\text{…}>\lambda_{\min}=0\right\}$ of the parameter λ using least angle regression (LARS, Efron et al., 2004). Function solveEN() finds solutions for the Elastic‐Net problem for given values of α and λ via the Coordinate Descent algorithm (Friedman et al., 2007).

The SFSI R‐package can be installed from CRAN using the following instruction:

install.packages(“SFSI”)

Alternatively, the package can be installed from the GitHub platform using the instructions below:

install.packages(“remotes”)

library(remotes)

install_github(“MarcooLopez/SFSI”)

The following snippet shows how to fit an Elastic‐Net regression using the SFSI R‐package. First, we simulate a sample data set (including predictors, X and a phenotype, y). Subsequently, we compute the sufficient statistics (Σ and Γ) and, finally, derive estimates of effects using the solveEN() function for a set of values of λ that are obtained using the LARS() function. For comparison, the weights are also computed with the glmnet() function of the glmnet (Friedman et al., 2010) R‐package.

# Libraries

library(SFSI); library(glmnet)

# Data simulation

n = 3000; p = 500

b = rep(0,p); b[sample(1:p, size = p*0.1)] = rgamma(p*0.1,4,4)

X = scale(matrix(rnorm(n*p), ncol = p))     # predictors matrix

signal = X %*% b

error = rnorm(n, sd = 3*sd(signal))

y = scale(signal + error)           # response vector

# Computing sufficient statistics

Sigma = crossprod(X)/n     # (co)variance of predictors

Gamma = crossprod(X,y)/n   # variance between predictors and response

# Fitting models using three different fuctions

fm0 = LARS(Sigma, Gamma, method = “LASSO”)   # find the entire lambda path

fm1 = solveEN(Sigma, Gamma, lambda = fm0$lambda)

fm2 = glmnet(X, y, lambda = fm0$lambda)

The functions LARS() and solveEN() return a matrix ($beta) with solutions for the coefficients ${\hat{\beta }}^{\left(\lambda \right)}$ (in rows) for each of the values of the regularization parameter (λ, in columns). The solutions provided by solveEN() and glmnet() are identical (up to a level of precision controlled by arguments “tol” and “thresh,” respectively). However, as noted, the fact that solveEN() and LARS() use Σ and Γ as inputs gives these functions of the SFSI R‐package great flexibility in terms of the problems that can be solved with it. For instance, this flexibility allows the SFSI R‐package to solve selection index problems.

### 2.2 Sparse selection indices for indirect phenotypic selection

An SI (Hazel, 1943; Smith, 1936) predicts the genetic value ($g_{i}$) of a selection candidate for a breeding objective ($y_{i}$) as a weighted sum of p indicator (i.e., measured) traits, $x_{i}={\left(x_{i1},\text{…},x_{\mathrm{ip}}\right)}^{\prime }$, as ${\hat{g}}_{i}=w^{\prime }x_{i}$. Here, we assume that these traits were adjusted by non‐genetic effects. In a standard SI the weights of the SI, $w={\left(w_{1},\text{…},w_{p}\right)}^{\prime }$, are derived by minimizing the mean squared prediction error, $\frac{1}{2}\mathrm{MSPE}=\frac{1}{2}\;E{\left(g_{i}-w^{\prime }x_{i}\right)}^{2}$. As detailed in Lopez‐Cruz et al. (2020), the MSPE function can be expressed as $\frac{1}{2}w^{\prime }P_{x}w-{G_{\mathrm{xy}}}^{\prime }w$, where $P_{x}$ is the phenotypic (co)variance matrix of the measured traits, and $G_{xy}$ is a vector with genetic covariances between the selection objective and each measured trait. The solution to this problem is $\hat{w}=P_{x}^{-1}\;G_{\mathrm{xy}}$.

The SI methodology was originally developed for problems involving a small number of measured traits. High‐throughput technologies can produce thousands of indicator traits (e.g., imaging, sensors, gene expression). This can cause overfitting leading to a low‐heritability index, which in turn leads to poor accuracy of indirect selection. To address this problem, we (Lopez‐Cruz et al., 2020) developed a SSI, which derives the weights using the following penalized MSPE optimization problem

(2) $$\hat{w}=\underset{w}{arg\;\min}\;\left[\frac{1}{2}w^{\prime }P_{x}w-w^{\prime }G_{\mathrm{xy}}+\lambda F\left(w\right)\right]$$

where $F\left(w\right)=\alpha \sum_{j=1}^{p}\left|w_{j}\right|+\frac{1}{2}\left(1-\alpha \right)\sum_{j=1}^{p}w_{j}^{2}$ is a sparsity‐inducing penalty function such as the one in Equation (1). This Equation (2) has the same form as that of Equation (1) with $\Sigma =P_{x}$ and $\Gamma =G_{\mathrm{xy}}$. Therefore, the coefficients of an SSI can be obtained as a penalized regression using solveEN() and LARS() functions. The steps needed to implement an SSI are as follows:

- Obtain estimates of $P_{x}$ and $G_{xy}$: The first matrix can be estimated using a method of moments, that is, ${\hat{P}}_{x}=\frac{1}{n}\;X^{\prime }X,$where $X=\{x_{\mathrm{ij}}\}$ is a matrix of centered indicator traits and n is the sample size. The vector $G_{\mathrm{xy}}={\left(G_{x_{1},y},\text{…},G_{x_{p},y}\right)}^{\prime }$ includes genetic covariances between the indicator traits and the selection objective. These can be estimated using a sequence of two‐trait genomic models fitted to the corresponding pairs of traits of traits (e.g., Butler et al., 2023; Covarrubias‐Pazaran, 2016; Pérez‐Rodríguez & de los Campos, 2022).
- Derive the weights ${\hat{w}}^{\left(\lambda \right)}$ using the solveEN() function by providing $P_{x}$ and $G_{xy}$: This is usually done over a grid of values of the regularization parameter (λ). By default, solveEN() runs the model over a grid of 100 possible values of λ (see Supporting Information S1).
- Evaluate the SI for the selection candidates, ${\hat{g}}^{\left(\lambda \right)}=X{\hat{w}}^{\left(\lambda \right)}$.
- Estimate the accuracy of each of the indices; the results can be used to select an optimal value of λ.

Once $P_{x}$ and $G_{xy}$ have been estimated, steps (ii) and (iii) can be executed using the following code:

# Px: (co)variance matrix (p x p) of measured traits

# Gxy: vector (p x 1) of genetic covariances between measured traits and response

# X: matrix of measured traits in selection candidates

fm = solveEN(Px, Gxy) # derivation of weights

gHat = predict(fm, X = X) # SI for the selection candidates

The first line of the script derives the weights for the SSI (the solution to Equation 2) using the solveEN() function, and the second line shows how to obtain predictions using the fitted model and a matrix containing the values of the indicator traits (individuals in rows, indicator traits in columns).

### 2.3 Sparse genomic prediction

In genomic prediction, the genomic BLUP (GBLUP; e.g., VanRaden, 2007, 2008) predicts the genetic merit of selection candidates (i.e., testing [TST]), ${\hat{g}}_{\mathrm{TST}}=\left\{{\hat{g}}_{\mathrm{TST}(i)}\right\}$ as a weighted sum of the phenotypic values of individuals in a training (TRN) set, $y_{\mathrm{TRN}}$. If there are no fixed effects (e.g., if the training phenotypes were adjusted by non‐genetic effects), GBLUP predictions can be written as (e.g., Henderson, 1975) ${\hat{g}}_{\mathrm{TST}}=Wy_{\mathrm{TRN}}$, where $W=G_{\mathrm{TRN},\mathrm{TST}}^{\prime }\;P_{\mathrm{TRN}}^{-1}$ is a $n_{\mathrm{TST}}\times n_{\mathrm{TRN}}$ matrix of weights with $G_{\mathrm{TRN},\mathrm{TST}}=\sigma_{u}^{2}\;K_{\mathrm{TRN},\mathrm{TST}}$ and $P_{\mathrm{TRN}}=\sigma_{u}^{2}\;K_{\mathrm{TRN}}+\sigma_{\varepsilon }^{2}I$. Here, $K_{\mathrm{TRN}}$ is a genomic relationship matrix (GRM) between individuals of the training set, $K_{\mathrm{TRN},\mathrm{TST}}$ is a GRM between the individuals of the training set and the candidates of selection, and $\sigma_{u}^{2}\;$ and $\sigma_{\varepsilon }^{2}$ are genetic and error variances, respectively. GBLUP predictions are equivalent to those of a standard SI where the “measured traits” are the training phenotypes (Lopez‐Cruz & de los Campos, 2021).

The presence of imperfect linkage disequilibrium between markers and causal loci, non‐additive effects, and population structure can make the GBLUP sub‐optimal. To deal with this, we proposed using a SGP (Lopez‐Cruz & de los Campos, 2021); an approach in which the prediction of the $i^{\mathrm{th}}$ individual of the testing set is a weighted sum of a subset of the data from the training set. In SGP, the sparsity of the weights $\hat{W}={\left({\hat{w}}_{1},\text{…},{\hat{w}}_{n_{\mathrm{TST}}}\right)}^{\prime }$ is achieved by solving for the weights using the following penalized objective function:

(3) $${\hat{w}}_{i}=\underset{w_{i}}{arg\;\min}\;\left[\frac{1}{2}w_{i}^{\prime }P_{\mathrm{TRN}}w_{i}-w_{i}^{\prime }G_{\mathrm{TRN},\mathrm{TST}\left(i\right)}+\lambda F\left(w_{i}\right)\right]$$

where $P_{\mathrm{TRN}}=\sigma_{u}^{2}\;K_{\mathrm{TRN}}+\sigma_{\varepsilon }^{2}I$ and $G_{\mathrm{TRN},\mathrm{TST}(i)}$ is the *i*th column of $G_{\mathrm{TRN},\mathrm{TST}}=\sigma_{u}^{2}\;K_{\mathrm{TRN},\mathrm{TST}}$. The weights derived from Equation (3) using a λ=0 correspond to those of the standard GBLUP.

The SGP problem in Equation (3) has the same structure as that of the SSI in Equation (2); therefore, it can also be solved as a penalized regression using the solveEN() (or LARS()) function by setting $\Sigma =P_{\mathrm{TRN}}$ and $\Gamma =G_{\mathrm{TRN},\mathrm{TST}(i)}$ in Equation (1). The steps needed to derive an SGP are as follows:

- Derive a genomic (or pedigree‐based) relationships, including those among the training set, $K_{\mathrm{TRN}}$, as well as the ones between training and testing genotypes, $K_{\mathrm{TRN},\mathrm{TST}}$.
- Estimate variances $\sigma_{u}^{2}$ and $\sigma_{\varepsilon }^{2}$.
- Compute $P_{\mathrm{TRN}}=\sigma_{u}^{2}\;K_{\mathrm{TRN}}+\sigma_{\varepsilon }^{2}I$ and $G_{\mathrm{TRN},\mathrm{TST}}=\sigma_{u}^{2}\;K_{\mathrm{TRN},\mathrm{TST}}$.
- Derive the weights ${\hat{w}}_{i}$ by solving the penalized problem in Equation (3) for all testing individuals ($i=1,..,n_{\mathrm{TST}}$) using $\Sigma =P_{\mathrm{TRN}}$ and $\Gamma =G_{\mathrm{TRN},\mathrm{TST}}$. Note that here Γ has $n_{\mathrm{TST}}$ columns, in this case, the solveEN() function returns the full $n_{\mathrm{TST}}\times n_{\mathrm{TRN}}$ matrix of weights $\hat{W}$. Likewise, weights ${\hat{W}}^{\left(\lambda \right)}$ are derived over a grid of values of the regularization parameter (λ).
- Derive predictions using ${\hat{g}}_{\mathrm{TST}}^{\left(\lambda \right)}={\hat{W}}^{\left(\lambda \right)}\;y_{\mathrm{TRN}}$. An optimal value of λ can be obtained by cross‐validation performed within training data (see Supplementary Information S2).

The function SGP() is a wrapper that can perform steps (iii)–(v) after K, $\sigma_{u}^{2}$ and $\sigma_{\varepsilon }^{2}$ have been obtained. Internally, this function calls the solveEN() function to fit all $n_{\mathrm{TST}}$ penalized regressions for specific testing individuals.

The following code illustrates how to use the SGP() function to derive predictions for a testing set using as arguments index vectors “trn” and “tst,” indicating which observations should be used for training and which ones should be used for testing, respectively.

# n: number of genotypes

# K: genomic relationship matrix (n x n)

# varU, varE: genetic and error variances

# y: vector of phenotypes of length n

# trn: vector indexing training set, e.g., trn = seq(1,0.8*n) first 80% of entries

# tst: vector indexing testing set, e.g., tst = seq(1,n)[‐trn] remaining 20% of entries

fm = SGP(K = K, varU = varU, varE = varE, trn = trn, tst = tst)

gHat = predict(fm, y = y) # predictions for the testing set

The first line of the code above derives the weights for the testing data using the variance parameters ($\sigma_{u}^{2}$ and $\sigma_{\varepsilon }^{2}$) and the GRM information ($K_{\mathrm{TRN}}$ and $K_{\mathrm{TRN},\mathrm{TST}}$). The second line derives the predictions for testing individuals using these weights and the phenotypes of the training set ($y_{\mathrm{TRN}}$). If the phenotypes are provided to the SGP() function, predictions for testing individuals are derived internally and provided in the output:

fm = SGP(y = y, K = K, varU = varU, varE = varE, trn = trn, tst = tst)

gHat = fm$yHat # predictions for the testing set

Optionally, if the variance parameters are not provided to the SGP() function, these parameters are estimated from training data using the corresponding phenotypes and GRM entries. In this case, the SGP() function calls the fitBLUP() function (included in the SFSI R‐package) which implements single‐trait mixed‐effects models with a single variance component (besides the error variance) using GEMMA (Zhou & Stephens, 2012). A standard GBLUP model can be fitted with this function using the training set as

fm = fitBLUP(y = y, K = K, trn = trn)

varU = fm$varU; varE = fm$varU # genetic and error variances

### 2.4 Multi‐trait/environment sparse genomic prediction

The SSI and SGP problems discussed previously derive predictions by borrowing information from indicator phenotypes measured in the candidate of selection and from the target phenotype measured in training genotypes. These two sources of information can be combined into a unified multi‐trait/environment prediction problem which enables borrowing of information between traits/environments and individuals.

Suppose we measure q phenotypes (either the same trait evaluated in several environments, different traits evaluated in a single environment, or several traits measured in several environments) and let $y_{k}$, $g_{k}$, and $\varepsilon_{k}$ to be vectors of phenotypes, genetic, and non‐genetic effects corresponding to the $k^{\mathrm{th}}$ phenotype (k=1,…,q). A unified prediction model can be fitted by considering $y={\left(y_{1}^{\prime },\text{…},y_{q}^{\prime }\right)}^{\prime }$, $g={\left(g_{1}^{\prime },\text{…},g_{q}^{\prime }\right)}^{\prime }$, and $\varepsilon =(\varepsilon_{1}^{\prime },\text{…},\varepsilon_{q}^{\prime })$ as the stacked vectors of phenotypes, genetic, and non‐genetic effects, respectively. If all phenotypes are measured on all individuals, then var(y)=(Ω⊗K)+(R⊗I), where Ω and R are q×q within‐subject genetic and error (co)variances matrices, respectively, and ⊗ denotes the Kronecker product. In cases where some phenotypes were not measured on some individuals, the (co)variance matrix of the observed phenotypes is a sub‐matrix of (Ω⊗K)+(R⊗I).

The weights of a multi‐trait/environment SGP (MT‐SGP) can be obtained by solving the objective function in Equation (3) with $P_{\mathrm{TRN}}=(R⊗K)\mathrm{TRN}+(R⊗I)\mathrm{TRN}$ and $G_{\mathrm{TRN},\mathrm{TST}}={\left(\Omega ⊗K\right)}_{\mathrm{TRN},\mathrm{TST}}\;$. Here, ${\left(.\right)}_{\mathrm{TRN}}$ and ${\left(.\right)}_{\mathrm{TRN},\mathrm{TST}}$ index subsets of the Kronecker product as training and prediction sets can encompass any genotype‐trait/environment combination from all possible ones (i.e., Kronecker product).

The required inputs to implement a multi‐trait/environment SGP (MT‐SGP) are the matrices K, Ω, and R. The following script describes how to derive such predictions for a problem involving q=3 phenotypes. The vectors “ID_geno” and “ID_trait” map entries of the phenotype vector to rows (and columns) of the GRM and traits (or environments, i.e., rows and columns of Ω and R). In the example below, we show a hypothetical situation with complete data where all three phenotypes were measured in all n individuals; however, incomplete data cases can be handled by appropriate specification of “ID_geno” and “ID_trait.”

# n: number of genotypes

# y1, y2, y3: vectors of phenotypes, each of length n

# Omega, R: genetic and error (co)variance matrices (3 × 3)

y = c(y1, y2,y3)    # stacked vector of phenotypes

ID_geno = rep(1:n, times = 3)    # vector indexing genotypes: 1,2,…,n,1,2,…,n,1,2,…,n

ID_trait = rep(1:3, each = n)    # vector indexing traits: 1,1,…,1,2,2,…,2,3,3,…,3

fm = SGP(y, K = K, varU = Omega, varE = R, trn = trn, tst = tst, ID_geno = ID_geno, ID_trait = ID_trait)

### 2.5 Datasets

To benchmark MT‐SGP against MT‐GBLUP, we used three crop data sets covering 30 traits/environments.

#### 2.5.1 Wheat dataset

This dataset is from CIMMYT's Global Wheat Program (Braun et al., 1996) and includes adjusted phenotypic records of grain yield (t ha^−1^) from n=3731 wheat (*Triticum aestivum*) lines evaluated at four environmental conditions (B2I, B5I, MEL, and LHT) and marker data for 9045 SNPs. This dataset is a subset, corresponding to the lines that have data in the four environments, from the full dataset described and analyzed by Pérez‐Rodríguez et al. (2017) and Lopez‐Cruz and de los Campos (2021).

#### 2.5.2 Maize dataset

The second dataset is from the Genomes‐to‐Fields project (Lawrence‐Dill et al., 2019) and comprises adjusted phenotypic records on four traits (grain yield [t ha^−1^], days‐to‐anthesis, anthesis‐silking interval, and plant height [cm]) from maize (*Zea mays*) hybrids evaluated at US North (n=4132) and South (n=1868) regions, and marker data on 98,026 SNPs. This dataset is a subset, corresponding to the hybrids that have data on the four traits, from the full dataset widely described and analyzed by Lima et al. (2023) and Lopez‐Cruz et al. (2023).

#### 2.5.3 Rice dataset

The third dataset is a worldwide collection from the Rice Diversity Project (www.ricediversity.org) and contains adjusted phenotypic records of n=413 rice (*Oryza sativa*) diversity lines on 20 traits classified into different categories (flowering, morphology, yield components, seed morphology, stress tolerance, and quality) and marker data on 25,085 SNPs. This dataset is described in detail and analyzed by Zhao et al. (2011).

### 2.6 Analyses

To evaluate the PA of the MT‐SGP and MT‐GBLUP, we performed prediction analysis using randomized training‐testing data partitions. For each dataset, we sampled testing data sets using the cross‐validation (CV) scheme CV2 (described in Burgueño et al. [2012]), in which the goal is to predict the performance of genotypes that have been evaluated for (in) some but not all the traits (environments). Specifically, for each training‐testing partition we randomly sampled $n_{0}$ (of the n available) genotypes to contribute data to a testing set, and for each of these genotypes, we assigned at random a pair of measurements (traits/environments) to form the testing data set. Therefore, the total number of data point forming each testing set was $n_{\mathrm{TST}}=n_{0}\times 2$ phenotypic records. The training data set included the remaining $n_{\mathrm{TRN}}=\left(n\times q\right)-n_{\mathrm{TST}}$ records. The number of genotypes contributing data to the testing set ($n_{0}$) was chosen such that all genotype‐trait/environment combinations were equally represented in the training and prediction set.

For each dataset, a GRM was obtained for the n genotypes from m SNP markers as $K={\mathrm{ZZ}}^{\prime }/v$, where $Z=\{z_{ij}\}$ is the n×m matrix of centered markers (VanRaden, 2007, 2008) and $v=\sum_{j=i}^{m}\mathrm{var}\left(z_{ij}\right)\;$ is a constant that makes the average diagonal value of K equal to one (Hayes et al., 2009). We used this GRM and the training phenotypes to derive predictions. The steps used to derive the MT‐SGP were as follows: First, we estimated the genetic (Ω) and error (R) (co)variance matrices from the training data set using the Multitrait() function of BGLR R‐package v1.1.0 (Pérez‐Rodríguez & de los Campos, 2022). With this function, we fitted Bayesian multi‐trait/environment models with genetic and environmental effects assumed to follow a multivariate Gaussian prior with unstructured (co)variance matrices (Ω and R). For the wheat dataset, because measurements were taken in different environments, R was assumed to be diagonal. Second, we derived the weights ${\hat{W}}^{\left(\lambda \right)}$ for the testing data using the GRM K, and matrices Ω and R. This was done using the SGP() function passing index vectors “trn” and “tst” defining training and testing sets, respectively. Third, we used the weights and the phenotypes of the training set, $y_{\mathrm{TRN}}$, to derive predictions of testing data as ${\hat{g}}_{\mathrm{TST}}^{\left(\lambda \right)}={\hat{W}}^{\left(\lambda \right)}\;y_{\mathrm{TRN}}$. This was done using the predict() function. The MT‐GBLUP was implemented using the above procedure with λ=0 in the second step.

Finally, we evaluated PA by correlating observed and predicted values in the testing set, that is, $\mathrm{cor}(y_{\mathrm{TST}},{\hat{g}}_{\mathrm{TST}}^{\left(\lambda \right)})$. These correlations were calculated within trait/environment using the corresponding $n_{\mathrm{TST}(k)}$ records (i.e., 1/q of the testing data) per trait/environment.

In the first set of analyses, we evaluated the PA as a function of the regularization parameter λ for a grid of 100 values of λ (see Supplementary Information S1). Then, we repeated the analyses using an optimal value ($\lambda_{\mathrm{CV}}$) of the parameter λ obtained by conducting a 10‐fold CV (Supplementary Information S2) within each training set ($n_{\mathrm{TRN}}$). The accuracy of the optimal MT‐SGP was also compared with that of the MT‐GBLUP (λ=0). We also derived the optimal single‐trait/environment SGP (ST‐SGP) and GBLUP (ST‐GBLUP) using the corresponding $n_{\mathrm{TRN}(k)}$ (i.e., 1/q of the training data) and the $n_{\mathrm{TST}(k)}$ records within each trait/environment (Equation 3). In total, we performed 100 training‐testing partitions estimating Ω and R at each partition; the results presented correspond to averages (and SDs) across such partitions.

The size of the training and testing sets, as well as the number of SNPs and phenotypes of each of the data sets are summarized in Table 1. The maize dataset was analyzed by region and in the rice dataset we stratified analyses by trait category.

**TABLE 1:** The Number of genotypes, traits (or environments), single‐nucleotide polymorphisms (SNPs), and phenotypic measurements, by data set.

Dataset		Genotypes	Traits	SNPs	Records		
					Training	Testing	Total
Wheat		3731	4	9045	10,448	4476	14,924
Maize	North	4132 1868	4	98,026	11,572	4956	16,528
	South				5240	2232	7472
Rice	Morphology	413	4	25,085	7780	480	8260
	Yield components		6		7540	720	8260
	Seed morphology		5		7660	600	8260
	Quality		3		7900	360	8260

#### 2.6.1 Software

The analyses were implemented using the SFSI R‐package v1.4.2. Calculation of between‐traits/environment genetic and error (co)‐variance matrices was performed using the BGLR R‐package v1.1.0 (Pérez‐Rodríguez & de los Campos, 2022). The scripts to perform all the analyses are provided in the Supporting Information Materials.

## 3 RESULTS

**FIGURE 1:**
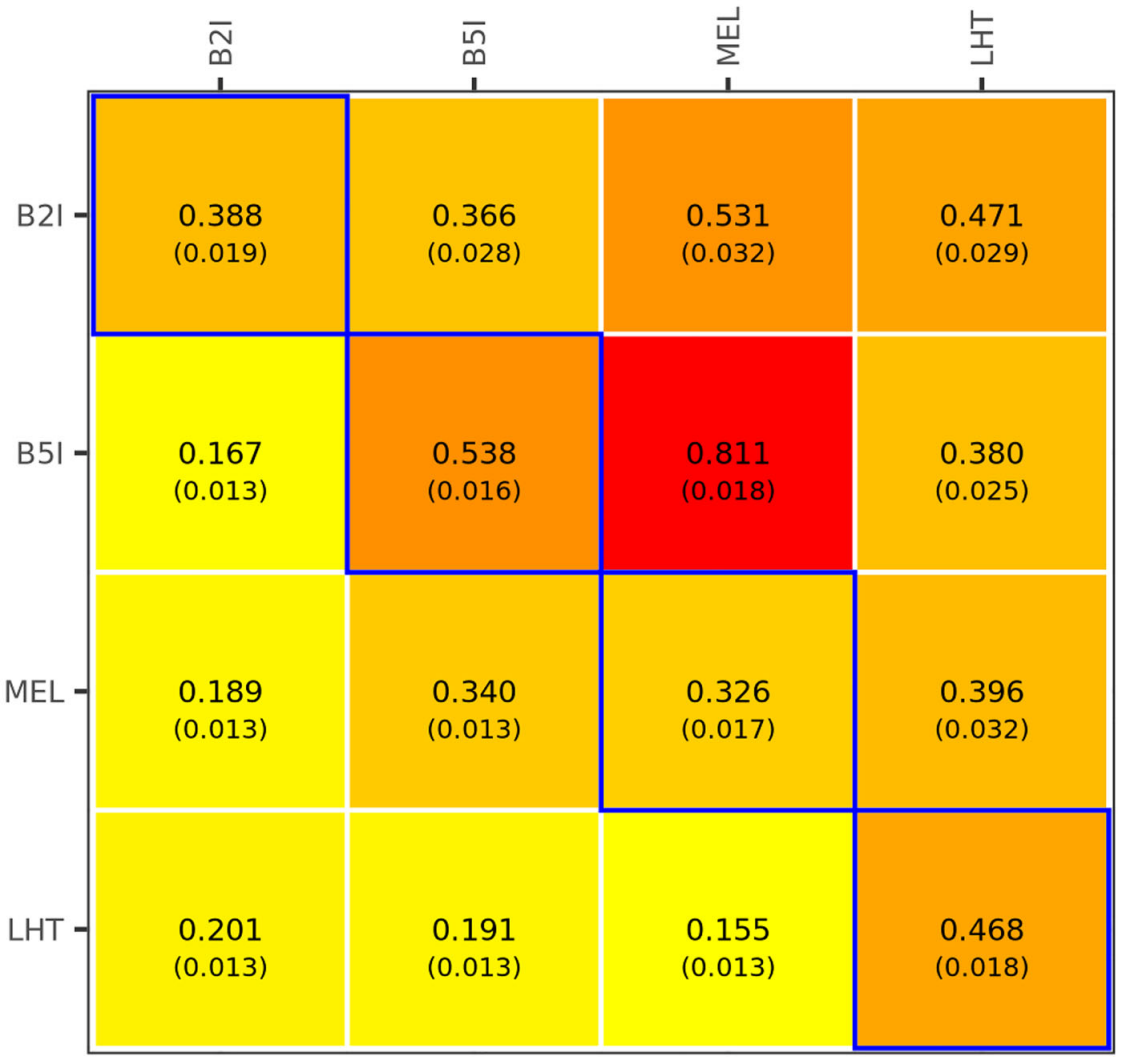
Genetic correlation (above the diagonal), phenotypic correlations (below the diagonal), and heritabilities (in the diagonal) of grain yield between environments (B2I, B5I, MEL, and LHT) for the wheat dataset (n=3731). B2I: bed planting + 2 irrigations, B5I: bed planting + 5 irrigations, MEL: flat planting + 5 irrigations, LHT: late planting date.

The heatmap in Figure 1 displays the estimated genetic correlations (upper triangle), phenotypic correlations (lower triangle), and heritabilities (diagonal) between environments for the wheat dataset. Grain yield showed moderately positive (∼0.37–0.53) genetic correlations between environments. Environments B5I and MEL stand by having a high genetic correlation (0.81 ± 0.019) because these environments have similar management conditions (same planting date and irrigation level; differing only in planting system). The maize and rice datasets showed a wider range of genetic correlations, ranging from low to moderate (∼0.02–0.63, Figure S1) and from low to high (∼0.01–0.86, Figure S2), respectively.

**FIGURE 2:**
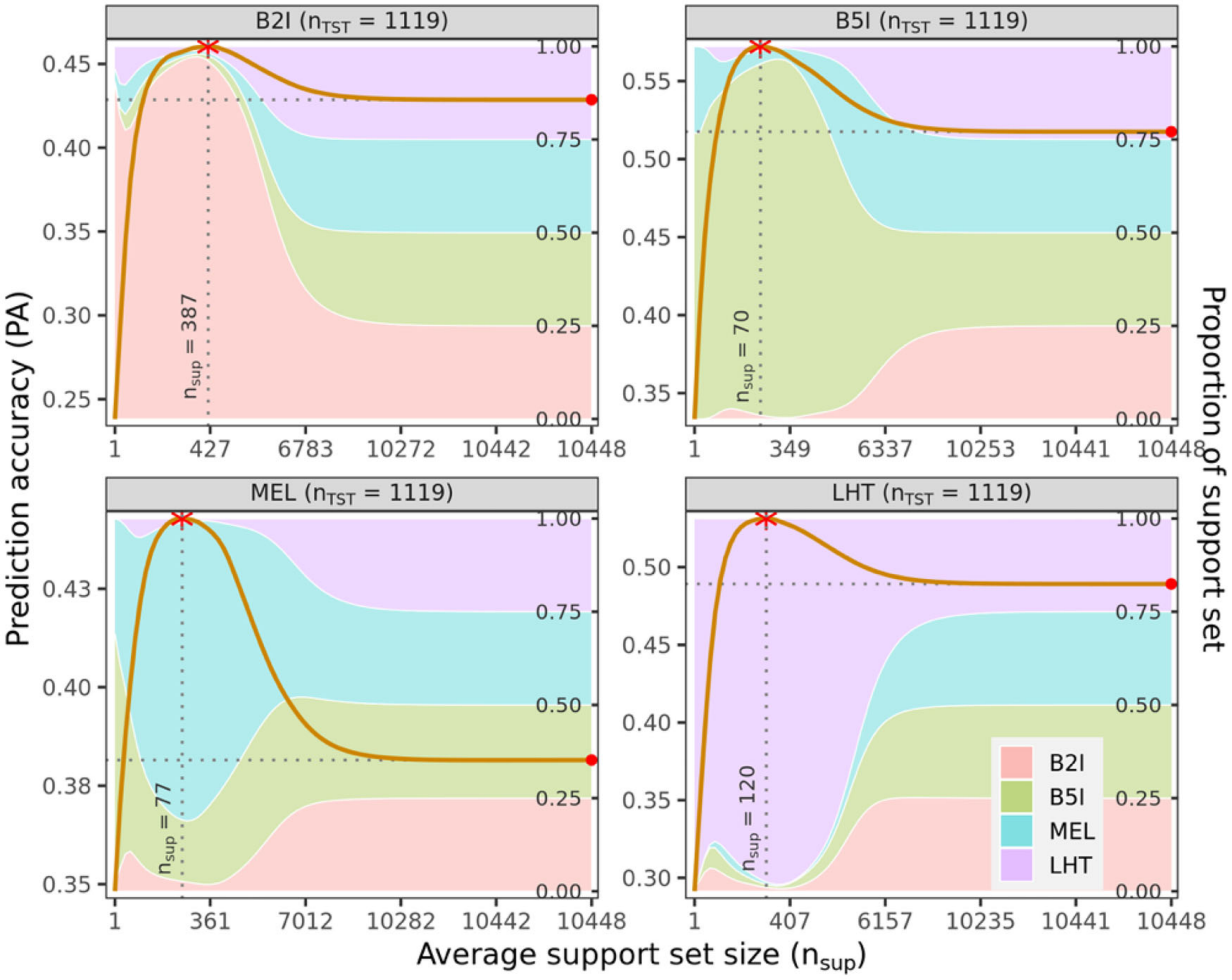
Within‐environment prediction accuracy (PA, average across 100 training‐testing partitions, represented by the solid curve) in testing data (CV2) of a multi‐trait‐sparse genomic prediction (MT‐SGP) versus the (average across training‐testing partitions) number of non‐zero weights of the prediction equation ($n_{\sup}(\lambda$)), in the wheat dataset (n=3731). The colored areas represent the proportion of non‐zero weights that corresponded to training set phenotypes collected in each of the environments. The red stars show the maximum PA achieved within each environment, and the solid points at the rightmost part of the curve mark the PA achieved by the multi‐trait‐genomic best linear unbiased prediction (MT‐GBLUP) (i.e., the MT‐SGP with λ=0).

Figure 2 shows the PA in testing data achieved by the MT‐SGP as a function of the sparsity of the solutions (i.e., $n_{\sup(\lambda )}$: the average number of training observations with a non‐zero weight for each value of the regularization parameter (λ) in the grid) by environment for the wheat dataset (see Figures S3 for the maize, and Figures S4–S7 for the rice datasets. These “area plots” can be generated using the multitrait.plot() function included in the SFSI R‐package). As reference, the rightmost points in the same plot correspond to the PA achieved by the MT‐GBLUP, in which all training observations have a non‐zero weight (i.e., the number of support points is equal $n_{\mathrm{TRN}}=10,448$). The highest PA achieved by the MT‐SGP (denoted with a star over the PA curve) happened with relatively high sparsity and was higher than the accuracy of the MT‐GBLUP in all the environments. The optimal MT‐SGP used, on average, 1%—5% of the training data ($n_{\sup(\lambda )}\approx$70‐387 of the 10,448 training data point available, Figure 2).

The colored bands in the background of each of the plots of Figure 2 represent the proportion of non‐zero weights that correspond to training data point collected in each of the environments. For the optimal MT‐SGP, the majority (but not all) of non‐zero weights correspond to observations collected within the same environment. As the penalty parameter was reduced and the MT‐SGP become less sparse, we see a more balanced contribution of data from all the environments to the prediction equations. Overall, these results suggest that a standard MT‐GBLUP weights too heavily on data from moderately correlated environments and that a more accurate prediction equation can be obtained using an MT‐SGP that borrow information primarily within environment and from genotypes that are closely related to the testing genotypes.

The shape of the PA curve achieved by the MT‐SGP suggests that with an adequate choice of the regularization parameter (λ), the MT‐SGP can offer gains in PA (relative to MT‐GBLUP) ranging from 7% (B2I) to 16% (MEL) for the wheat data set (Figure 2), 2%–6% for the maize data set (Figure S3), and 1%‐4% for the rice (Figures S4–S7) data set. However, those gains, chosen using the results in Figure 2 (or Figures S3, S4–S7), may be overestimated because, implicitly, an optimal value of λ is being selected by evaluating PA in testing data. To avoid this problem, we performed a similar prediction correlation‐versus‐λ profiling using only training data in a cross‐validation scheme (see Supplementary Information S2). Then, once an optimal λ was selected, we re‐fitted the MT‐SGP to the entire training set using the chosen $\lambda_{\mathrm{CV}}$ and, finally, we quantified the PA of the resulting MT‐SGP in testing data that was not used to choose λ. We also did this for the ST‐SGP.

**FIGURE 3:**
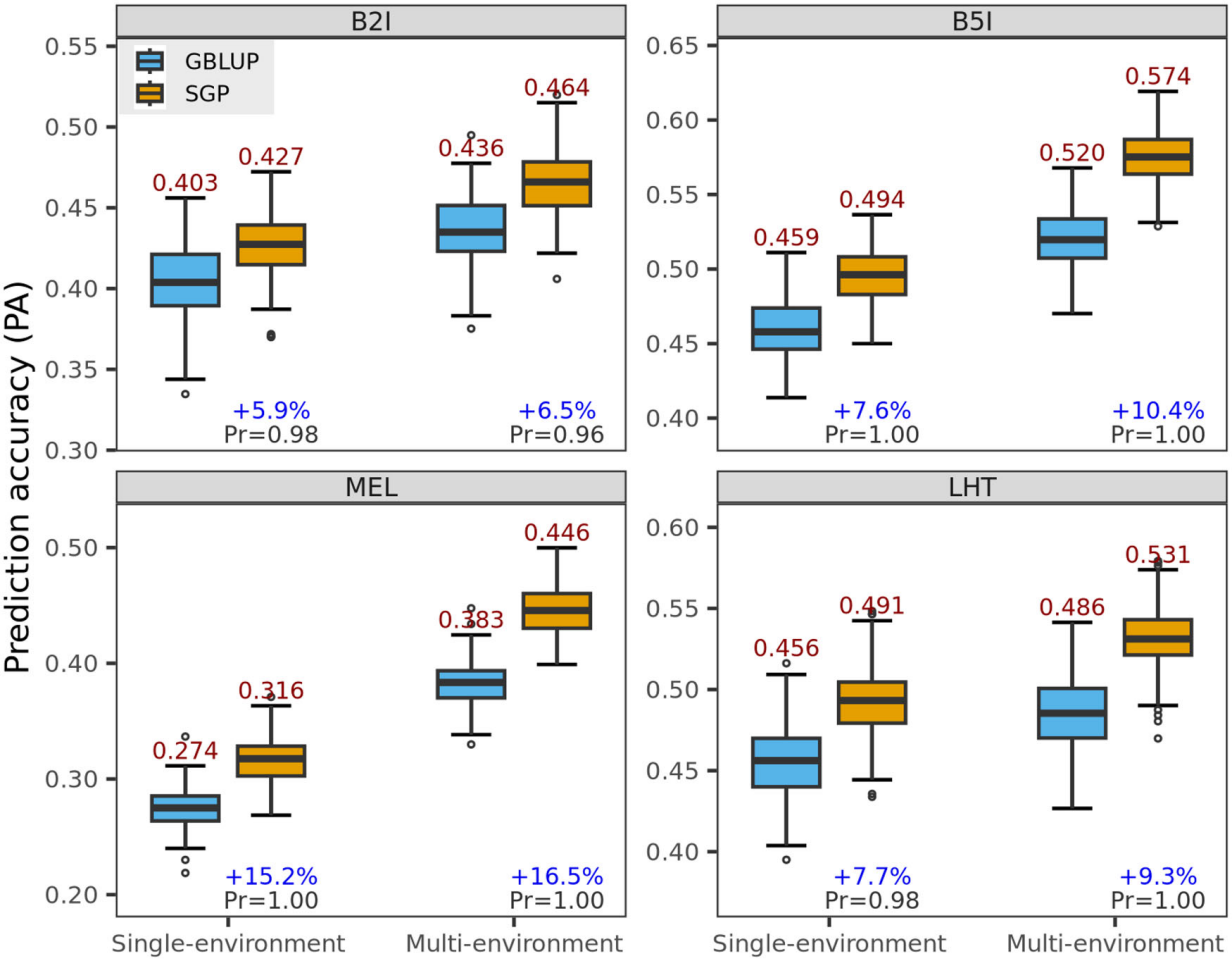
Within‐environment prediction accuracy (PA, average across 100 training‐testing partitions) achieved in single‐ and multi‐environment analysis using genomic best linear unbiased prediction (GBLUP) and sparse genomic prediction (SGP), in the wheat data set (n=3731). The percentage (in blue) indicates the gain in PA of the SGP over the GBLUP, and *Pr* indicates the proportion of times that the PA of the optimal SGP was higher than that of the GBLUP.

When the SGP model was fitted using a regularization parameter chosen through CV (internal to the training set), it outperformed the corresponding GBLUP models by margins ranging from 6% to 17% in the wheat data set (Figure 3), on average, 9% in ST models and 11% in MT models (Table S1). In the maize and rice data sets, the average gains were more moderate ranging from 0% to 6% (Figure S8) and 0.5%–3% (Figures S9–S12), respectively. The only exception to these patterns was the morphological trait in the rice data set where the SGP models did worse than the corresponding GBLUP models (Figures S9 and S13), with an average reduction in accuracy of 0.7% in ST and 3% in MT models (Table S1).

We assessed the bias of the predictions of the MT‐SGP and MT‐GBLUP. The bias was evaluated as the mean and slope of the regression of observed values on the predicted values, within trait/environment by dataset. The results show that there is a small increase in bias of the SGP (slope of 1.05–1.40) relative to GBLUP (slope of 1.01–1.21), suggesting that SGP add extra shrinking to the predictions (see Table S2).

### 3.1 Computational considerations

The derivation of SGP equations involves two steps: (i) estimation of (co)variance components and (ii) derivation of the weights of the prediction equations. The first step is also required for GBLUP prediction, and the computational time it takes to estimate (co)variance parameters depends on the software and estimation method used (see, for example, Pérez‐Rodríguez and de los Campos (2022) for computational benchmarks for the Multitrait() function of the BGLR R‐package). Therefore, here, we focus on the computational time involved in the second step, which is implemented using the SFSI R‐package.

The coordinate descent algorithm (Friedman et al., 2007) implemented in the solveEN() function obtains solutions using an iterative procedure. The algorithm stops when the maximum change in the values of the coefficients between the current and the previous iteration is below a tolerance value (parameter “tol”) or when a maximum number of iterations have been performed (parameter “maxiter”), whichever happens first. The computational time of solving one penalized regression is mainly affected by the number of iterations of the algorithm, which depends on the training set size and the value of λ.

We benchmarked the computational time required to derive an ST‐SGP (Equation 3) for a single testing genotype using training data sizes ($n_{\mathrm{TRN}}$) ranging from 1000 to 50,000. This was done for two values of λ: a large one producing a highly sparse solution (5% of the training data point having non‐zero weights) and a smaller one producing a solution with 10% of non‐zero weights. The matrices ($\Sigma =\sigma_{u}^{2}\;K_{\mathrm{TRN}}+\sigma_{\varepsilon }^{2}I$ and $\Gamma =\sigma_{u}^{2}\;K_{\mathrm{TRN},\mathrm{TST}(1)}$) used as inputs were sampled from a GRM obtained using a related wheat dataset from Lopez‐Cruz et al. (2022) with $\sigma_{u}^{2}=0.4$ and $\sigma_{\varepsilon }^{2}=0.6$. (This dataset is an extended collection of n=68,836 genotypes in a single environment, corresponding to environment B5I in the wheat dataset used here in the SGP analyses.) For each scenario, we performed 350 benchmarks, each time resampling the entries of the GRM used. The benchmarks were run in R v4.1.1 (R Core Team, 2021) compiled with the MKL's BLAS (v2020.4.304) running on the high‐performance computing cluster from Michigan State University (https://docs.icer.msu.edu/Cluster_Resources/) with Intel processors (Intel Xeon Gold 6148 CPU at 2.4 GHz, 96 GB of RAM) using either 2, 3, or 4 computing threads.

**FIGURE 4:**
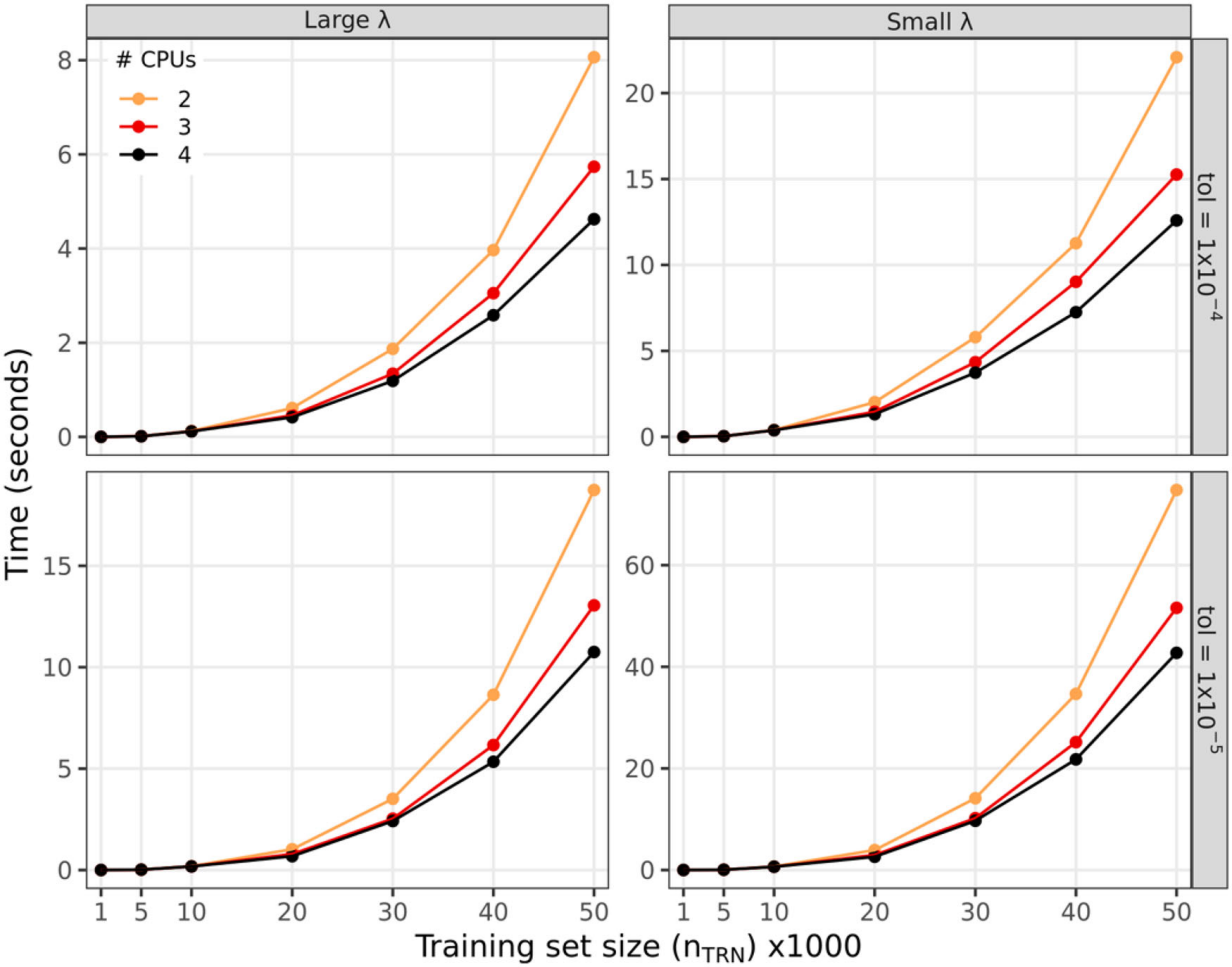
Computational time (in seconds) used by the solveEN() function to derive the weights of a single‐trait sparse genomic prediction (SGP) (Equation 3) for a single testing genotype for different training set sizes ($n_{\mathrm{TRN}}$), by value of the penalty parameter (λ, columns), tolerance error used to declare convergence (parameter “tol,” in rows), and number of CPUs (lines).

The computational time required to solve an SGP grew exponentially with the size of the training data set (Figure 4); however, the rate at which the computational time grows depends on the sparsity of the solution and on the tolerance used to declare convergence (Figure 4). As expected, a penalized regression with large values of λ (leading to highly sparse solutions) has a much lower computational cost than solutions that are less sparse (i.e., those with small values of λ). For instance, solving one ST‐SGP with $n_{\mathrm{TRN}}=40,000$ training individuals and a tolerance error of $1\;\times \;10^{-4},$ took ∼2.5–4 s for a large λ, and around 7–11 s for small λ (see top panels in Figure 4). Moreover, lowering the tolerance error requires much more iterations which is reflected in a larger computing time (Figure 14, compare top versus bottom panels in Figure 4).

We also benchmarked the computational time needed to derive an MT‐SGP for a single testing genotype, varying the number of training genotypes (n) and number of traits (q). This was done for a highly sparsity‐inducing value of λ and a tolerance error of $1\;\times \;10^{-4}$ using three computing threads. As before, the computational time needed to solve for one MT‐SGP increases with the number of genotypes and traits available for model training (Figure S15). On average, it takes ∼6 s to derive the weights for a single MT‐SGP when the number of training genotypes was 5000 and the number of traits was 10 (i.e., a total of 50,000 training data point). However, it took half of that time when the same number of data point originated from 50,000 individual genotypes that had measurements for a single trait (Figure S15).

## 4 DISCUSSION

In recent years, we have witnessed a continued increase in the size of genomic data sets, which have expanded both the number of genotypes as well as in the number of phenotypes (traits or environments) available for each training genotype. The continued increase in the size of the data sets available offers great opportunities to further advance genomic PA. However, from a prediction perspective, not all the data points included in modern genomic multi‐trait/environment are equally relevant, and some studies have indicated that sometimes using subsets of the available data may be preferable (e.g., Wolc et al., 2016). This has led to many investigations aiming to identify subsets of the data that are more relevant for a particular prediction problem.

For instance, many studies have evaluated the use of variable selection models to develop phenotypic prediction equations for economically relevant traits using high‐throughput phenotyping data in plants (e.g., Aguate et al., 2017; Montesinos‐López et al., 2017) and animals (e.g., Ferragina et al., 2015). However, these approaches rely on phenotypic covariances and, therefore, produce equations that do not necessarily deliver optimal genetic PA. The SSI methodology introduced by Lopez‐Cruz et al. (2020) addresses this problem by integrating selection index methodology and penalized regression, leading to prediction equations that maximize selection accuracy.

Likewise, there have been many investigations in genomic selection aiming at training set optimization, which consists of selecting, from a set of candidate genotypes, a subset of training individuals that is more relevant to a particular testing set. The goal of training set optimization is to keep the PA as big as possible while reducing the training set size, potentially allowing for lower phenotyping costs (Fernández‐González et al., 2023; Isidro y Sánchez & Akdemir, 2021; Rio et al., 2022). Examples of these methods include model‐based approaches (e.g., mean coefficient of determination, CDmean, and mean prediction error variance, PEVmean) (Akdemir et al., 2015; Akdemir & Isidro‐Sanchez, 2019; Isidro et al., 2015; Rincent et al., 2012) and model‐free approaches (e.g., average genomic relationship, avgGRM, partitioning around medoids, PAM, and fast and unique representative subset selection, FURS) (Atanda et al., 2021; Guo et al., 2019).

Building on the ideas used to develop SSIs, we introduced methodology for ST‐SGP (Lopez‐Cruz & de los Campos, 2021). SGP leads to prediction equations that are sparse in which only a subset of the training genotypes (i.e., those with non‐zero weight) contributes to the prediction of each of the testing genotypes.

### 4.1 SGP as a training set optimization technique

Although we developed SGP primarily as a prediction technique, it could potentially be used for training set optimization by choosing a subset of the training data. Indeed (like training set optimization methods), the derivation of the weights of prediction equations in SGP only require access to genomic relationships and regularization parameters (genetic and environmental (co)variances, and λ). For instance, one could imagine using the weights of a SGP model, derived before phenotypes were measured, to identify genotypes worth phenotyping (e.g., those with the largest frequency of non‐zero weight across all prediction equations). However, the use of SGP for training set optimization needs to be further investigated because, unlike standard training set optimization techniques, SGP does not assume that a single training set is optimal for all the candidates of selection. Instead, these methods produce prediction equations, each of which is potentially sparse but may not lead to the identification of a smaller training set because the training genotypes that do not contribute to the prediction equations of a selection candidate may still have a non‐zero weight in the prediction equation of other selection candidates. Likewise, in the multi‐trait/environment case, the MT‐SGP could be used to identify candidate genotype‐trait/environment combinations to be phenotyped.

The SSI and SGP methodologies can be used to derive prediction equations that borrow information from correlated traits and correlated genotypes, respectively. In this study, we present an MT‐SGP model that integrates SSI (Lopez‐Cruz et al., 2020) and SGP (Lopez‐Cruz & de los Campos, 2021) in a unified framework. The MT‐SGP model generates prediction equations that enables borrowing of information between traits and genotypes. Along with this methodology, we present the SFSI R‐package, which offers functionality to solve for SSIs, SGP, and MT‐SGP.

We benchmarked MT‐SGP against MT‐GBLUP suing three crop data sets covering 30 traits/environments. Overall, we found that MT‐SGP either outperforms MT‐GBLUP or, in some traits and populations, achieves the same PA as that of GBLUP (Figure S13). When the MT‐SGP outperforms MT‐GBLUP, it does it using highly sparse prediction equations that have non‐zero weights primarily for observations of the target phenotype/environment measured in genotypes that are genetically close to testing genotypes, as well as some information from traits/environments that are highly correlated with the target trait. Thus, if there is scope to improve prediction performance by selecting subsets of the training data set, MT‐SGP can identify those sets. When such selection is not needed or beneficial, for instance, if sample size is small, the MT‐SGP converges to MT‐GBLUP, thus achieving a comparable level of accuracy.

In general, the MT‐SGP and MT‐GBLUP outperformed the single‐trait/environment counterparts. This was expected because a multi‐trait/environment analysis offers opportunities to borrow information across correlated traits/environments (Burgueño et al., 2012; Calus & Veerkamp, 2011). However, the traits that benefited the most from multi‐trait/environment and sparse prediction were those that have moderate or low heritability and are genetically correlated with traits that have moderately high heritability (Jia & Jannink, 2012). This was particularly clear in the wheat data set, where the largest gains in accuracy (∼16%) were observed for the MEL environment (which has moderate heritability of 0.33 and high genetic correlation with B51 of 0.81, Figure 1). On the other hand, the advantage of using multi‐trait analyses were not as important in the maize and rice data set (Figures S8–S12) because of the lower genetic correlations between traits (Figures S1 and S2).

For some breeding problems, considering the bias of predictions may also be relevant. Therefore, we investigated possible mean and slope biases in the predictions of the MT‐SGP and MT‐GBLUP. The results suggest that SGP may lead to a small increase in slope bias relative to GBLUP, which suggests that the SGP may be over‐shrinking predictions. It is worth noting that in our study we selected the sparsity parameter to maximize prediction correlation between predictions and observations in testing data, which does not account for mean and slope bias. If slope bias is of concern, one could possibly reduce the bias by selecting the sparsity parameter by minimizing prediction mean squared error.

The computational time required to solve for SGP equations is typically higher than that of the GBLUP unless the solution is extremely sparse. However, the solution to SGP (and MT‐SGP) equations for many testing genotypes is an embarrassingly parallel task (the coefficients of the SGP of different testing genotypes can be solved independently). The computational cost to derive an SGP for a single testing genotype is mainly affected by the training data size, the sparsity of the solution, and the convergence tolerance. For multi‐trait implementation, the computational time grows with the number of traits/environments with a growing rate that is bounded by the number of unique genotypes in the training set rather than the total training set size.

In this study we focused on additive models; however, SGP can also be implemented using non‐additive models using parametric (e.g., models accounting for dominance or epistatic effects; Alves et al., 2019; Technow et al., 2012) or semi‐parametric (e.g., Reproducing Kernel Hilbert Spaces Regression; de los Campos et al., 2010; Gianola & Van Kaam, 2008) methods. The evaluation of SGP using non‐additive models appears as a natural area of future research. Likewise, the evaluation of SGP for large multi‐environment data (e.g., Lima et al., 2023) as well as the use of SGP for models integrating SNPs and environmental covariates (Jarquín et al., 2014; Lopez‐Cruz et al., 2023) seems a promising research area. Finally, although we have optimized the implementation of the coordinate descent algorithm using state‐of‐the‐art approaches, there are still opportunities to speed up the derivation of SGP equations that we aim to pursue in future releases of the SFSI R‐package.

## 5 CONCLUSIONS

We introduced a novel MT‐SGP model that combines the features of SSIs and SGP and presented an R‐package that can be used to solve those problems. The MT‐SGP model can be used to derive prediction equations that borrow information from a subset of the candidates of selection and a subset of the measured traits/environments. Using real data, we show that the MT‐SGP either performs similarly or, in many traits/environments and populations, outperforms the MT‐GBLUP. The potential of the MT‐SGP to deliver gains in PA relative to MT‐GBLUP is largely influenced by the genetic structure of the data, sample size, trait heritability, and genetic correlations. Large data sets from structured populations involving groups of traits/phenotypes with moderate heritability that are highly genetically correlated seem to be the ones for which using MT‐SGP is more promising.

## AUTHOR CONTRIBUTIONS

**Marco Lopez‐Cruz**: Conceptualization; data curation; formal analysis; methodology; software; validation; writing—original draft; writing—review and editing. **Gustavo de los Campos**: Conceptualization; investigation; methodology; resources; supervision; visualization; writing—original draft; writing—review and editing.

## CONFLICT OF INTEREST STATEMENT

The authors declare no conflicts of interest.

## Supporting information



Supplemental Material

## Data Availability

The full wheat datasets used for the analyses include phenotypic and marker data and can be downloaded from CIMMYT's repositories (https://www.cimmyt.org/). The subset of the wheat data used in this study was deposited at the Dryad repository at https://doi.org/10.5061/dryad.vx0k6dk3p. The maize data set is available at the Figshare repository at https://doi.org/10.6084/m9.figshare.22776806. The rice data set can be downloaded from the Rice Diversity repository (www.ricediversity.org/44kgwas). The data used for the computational benchmarks was taken from Lopez‐Cruz et al. (2022), and it is available at https://hdl.handle.net/11529/10548635.
