# Supplementary material for: Multi‐trait/environment sparse genomic prediction using the SFSI R‐package
Source: Plant Genome. 2025 Jun 13;18(2):e70050. doi: 10.1002/tpg2.70050 (PMC12166114; doi:10.1002/tpg2.70050)
Supplement: Supplementary file 1 [file TPG2-18-e70050-s001.docx]

**Supplemental Material**

Multi-trait/environment Sparse Genomic Prediction

using the SFSI R-package

Marco Lopez-Cruz and Gustavo de los Campos

**This file contains:**

- Supplemental Figure S1-S15.
- Supplemental Table S1-S2.
- Supplemental Note S1-S2.
- Supplemental Boxes S1-S11.

1. **Supplemental Figures**

**Supplemental Figure S1.** Genetic correlations (above the diagonal), phenotypic correlations (below the diagonal), and heritabilities (in the diagonal) between traits, in the north ($n=4132$) and south ($n=1868$) regions of the **maize dataset**. The estimates displayed are posterior means and posterior standard deviations (in parenthesis). Yield: grain yield, DTA: days-to-anthesis, ASI: anthesis-silking interval, PH: plant height

**Supplemental Figure S2.** Genetic correlations (above the diagonal), phenotypic correlations (below the diagonal), and heritabilities (in the diagonal) between traits in the **rice dataset** ($n=413$, $q=20$ traits), separated by trait category (flowering, morphology, yield components, seed morphology, and quality). The estimates displayed are posterior means and posterior standard deviations (in parenthesis). Pan.num.per.plant: panicle number per plant, Plant.H: plant height, Panicle.L: panicle length, Prim.pan.br.num: primary panicle branch number. Seed.num.per.pan: seed number per panicle, Florets.per.pan: florets per panicle

**Supplemental Figure S3.** Within-trait prediction accuracy (PA, average across 50 training-testing partitions) in testing data (CV2) of a MT-SGP versus the (average across training-testing partitions) number of non-zero weights of the prediction equation ($n_{\sup}(\lambda$)), in the north ($n=4132$) and south ($n=1868$) regions of the **maize dataset**. The colored areas represent the proportion of non-zero weights that corresponded to training-set observations collected in each of the traits. The red stars show the maximum PA achieved within each trait, and the solid points at the rightmost part of the curve mark the PA achieved by the MT-GBLUP (i.e., the MT-SGP with $\lambda=0$)

**Supplemental Figure S4.** Within-trait prediction accuracy (PA, average across 50 training-testing partitions) in testing data (CV2) of a MT-SGP versus the (average across training-testing partitions) number of non-zero weights of the prediction equation ($n_{\sup}(\lambda$)), for the **morphology** traits, **rice dataset** ($n=413$, $q=20$ traits). The colored areas represent the proportion of non-zero weights that corresponded to training-set observations collected in each of the traits. The red stars show the maximum PA achieved within each trait, and the solid points at the rightmost part of the curve mark the PA achieved by the MT-GBLUP (i.e., the MT-SGP with $\lambda=0$)

**Supplemental Figure S5.** Within-trait prediction accuracy (PA, average across 50 training-testing partitions) in testing data (CV2) of a MT-SGP versus the (average across training-testing partitions) number of non-zero weights of the prediction equation ($n_{\sup}(\lambda$)), for the **yield components** traits, **rice dataset** ($n=413$, $q=20$ traits). The colored areas represent the proportion of non-zero weights that corresponded to training-set observations collected in each of the traits. The red stars show the maximum PA achieved within each trait, and the solid points at the rightmost part of the curve mark the PA achieved by the MT-GBLUP (i.e., the MT-SGP with $\lambda=0$)

**Supplemental Figure S6.** Within-trait prediction accuracy (PA, average across 50 training-testing partitions) in testing data (CV2) of a MT-SGP versus the (average across training-testing partitions) number of non-zero weights of the prediction equation ($n_{\sup}(\lambda$)), for the **seed morphology** traits, **rice dataset** ($n=413$, $q=20$ traits). The colored areas represent the proportion of non-zero weights that corresponded to training-set observations collected in each of the traits. The red stars show the maximum PA achieved within each trait, and the solid points at the rightmost part of the curve mark the PA achieved by the MT-GBLUP (i.e., the MT-SGP with $\lambda=0$)

**Supplemental Figure S7.** Within-trait prediction accuracy (PA, average across 50 training-testing partitions) in testing data (CV2) of a MT-SGP versus the (average across training-testing partitions) number of non-zero weights of the prediction equation ($n_{\sup}(\lambda$)), for the **quality** traits, **rice dataset** ($n=413$, $q=20$ traits). The colored areas represent the proportion of non-zero weights that corresponded to training-set observations collected in each of the traits. The red stars show the maximum PA achieved within each trait, and the solid points at the rightmost part of the curve mark the PA achieved by the MT-GBLUP (i.e., the MT-SGP with $\lambda=0$)

**Supplemental Figure S8.** Within-trait prediction accuracy (PA, average across 50 training-testing partitions) achieved in single- and multi-trait models using GBLUP and Sparse Genomic Prediction (SGP), in the north ($n=4132$) and south ($n=1868$) regions of the **maize dataset**. The percentage (in blue) indicates the gain in PA of the SGP over the GBLUP, and *Pr* indicates the proportion of times that the PA of the optimal SGP was higher than that of the GBLUP. Yield: grain yield, DTA: days-to-anthesis, ASI: anthesis-silking interval, PH: plant height

**Supplemental Figure S9.** Within-trait prediction accuracy (PA, average across 50 training-testing partitions) achieved in single- and multi-trait models using GBLUP and Sparse Genomic Prediction (SGP), for the **morphology** traits in the **rice dataset** ($n=413$, $q=20$ traits). The percentage (in blue) indicates the gain in PA of the SGP over the GBLUP, and *Pr* indicates the proportion of times that the PA of the optimal SGP was higher than that of the GBLUP

**Supplemental Figure S10.** Within-trait prediction accuracy (PA, average across 50 training-testing partitions) achieved in single- and multi-trait models using GBLUP and Sparse Genomic Prediction (SGP), for the **yield components** traits in the **rice dataset** ($n=413$, $q=20$ traits). The percentage (in blue) indicates the gain in PA of the SGP over the GBLUP, and *Pr* indicates the proportion of times that the PA of the optimal SGP was higher than that of the GBLUP

**Supplemental Figure S11.** Within-trait prediction accuracy (PA, average across 50 training-testing partitions) achieved in single- and multi-trait models using GBLUP and Sparse Genomic Prediction (SGP), for the **seed morphology** traits in the **rice dataset** ($n=413$, $q=20$ traits). The percentage (in blue) indicates the gain in PA of the SGP over the GBLUP, and *Pr* indicates the proportion of times that the PA of the optimal SGP was higher than that of the GBLUP

**Supplemental Figure S12.** Within-trait prediction accuracy (PA, average across 50 training-testing partitions) achieved in single- and multi-trait models using GBLUP and Sparse Genomic Prediction (SGP), for the **quality** traits in the **rice dataset** ($n=413$, $q=20$ traits). The percentage (in blue) indicates the gain in PA of the SGP over the GBLUP, and *Pr* indicates the proportion of times that the PA of the optimal SGP was higher than that of the GBLUP

**Supplemental Figure S13.** Prediction accuracy of the GBLUP (x-axis) versus that of the optimal SGP (y-axis) in single- and multi-trait/environment, by dataset. Each point represents a training-testing partition (a total of 100 partitions) and colors represent either environment (wheat dataset), trait (maize dataset) or trait-group (rice dataset). The optimal SGP was obtained with a value $\lambda_{\mathrm{CV}}$ calculated using cross-validation within training sets. B2I: bed planting + 2 irrigations, B5I: bed planting + 5 irrigations, MEL: flat planting + 5 irrigations, LHT: late planting date, Yield: grain yield, DTA: days-to-anthesis, ASI: anthesis-silking interval, PH: plant height

**Supplemental Figure S14.** Number of iterations used by the solveEN() function to derive the weights of a single-trait SGP for a single testing genotype for different training set sizes ($n_{\mathrm{TRN}}$), by tolerance error used to declare convergence (parameter ‘tol’, in panels), and value of the penalty parameter ($\lambda$, lines). The matrices ($\boldsymbol{\Sigma=}\sigma_{u}^{2}\mathbf{K}_{\mathrm{TRN}}+\sigma_{\varepsilon}^{2}\mathbf{I}$ and $\boldsymbol{\Gamma=}\sigma_{u}^{2}\mathbf{K}_{TRN,TST(1)}$) used as inputs were sampled from a GRM obtained using data from Lopez-Cruz *et al.* (2022) with $\sigma_{u}^{2}=0.4$ and $\sigma_{\varepsilon}^{2}=0.6$. (This data set is an extended collection of $n=68,836$ genotypes.) For each scenario we performed 350 benchmarks, each time resampling the entries of the GRM used

**Supplemental Figure S15.** Computational time (in seconds) used by the solveEN() function to derive the weights of a MT-SGP for a single testing genotype for different training set sizes ($n_{\mathrm{TRN}}$), using a large value of $\lambda$ (producing 5% of the training data points having non-zero weights) and a tolerance error parameter ‘tol=1E-4’. The input matrices ($\boldsymbol{\Sigma=}\boldsymbol{(\Omega\otimes K)}_{\mathrm{TRN}}+\left( \boldsymbol{R\otimes I} \right)_{\mathrm{TRN}}$ and $\boldsymbol{\Gamma=}\boldsymbol{(\Omega\otimes K)}_{TRN,TST(1)}$) were derived by sampling from a GRM obtained using data from Lopez-Cruz *et al.* (2022), and $\boldsymbol{\Omega=}0.4\boldsymbol{\times\Phi}(0.7)$ with $\boldsymbol{\Phi}(\rho)$ a first-order autoregressive process AR(1) with autocorrelation $\rho=0.7$; and $\mathbf{R=}0.6\times\mathbf{I}$. Solid (blue) line represents the total training size given by the product of the number of genotypes ($n$, panels) and number of traits ($q$, top x-axis), $n_{\mathrm{TRN}}=n\times q$. Dotted line represents the case when the training set is composed of $n_{\mathrm{TRN}}$ different genotypes and a single trait.

2. **Supplemental Tables**

**Supplemental Table S1.** Average [min, max] (across traits/environments-partitions) prediction accuracy (PA) of the optimal SGP (obtained with a value $\lambda_{\mathrm{CV}}$ calculated using cross-validation within training sets) and GBLUP, and relative gain of the SGP over the GBLUP in single- and multi-trait/environment models, by dataset

| **Dataset** | | **Traits** | **Single-trait PA** | | | **Multi-trait PA** | | |
| --- | --- | --- | --- | --- | --- | --- | --- | --- |
|  |  |  | **GBLUP** | **SGP** | **Gain (%)** | **GBLUP** | **SGP** | **Gain (%)** |
| Wheat | | 4 | 0.40  [0.27,0.46] | 0.43  [0.32,0.49] | 9.1  [5.9,15.2] | 0.46  [0.38,0.52] | 0.50  [0.45,0.57] | 10.7  [6.5,16.5] |
| Maize | North | 4 | 0.72  [0.57,0.91] | 0.74  [0.59,0.91] | 2.5  [0.5,6.9] | 0.73  [0.58,0.91] | 0.74  [0.59,0.91] | 1.8  [-0.0,4.7] |
|  | South | 4 | 0.60  [0.34,0.81] | 0.60  [0.34,0.81] | 0.5  [-0.2,2.5] | 0.61  [0.35,0.82] | 0.61  [0.35,0.82] | 0.6  [-0.5,2.3] |
| Rice | Morphology | 4 | 0.59  [0.39,0.74] | 0.59  [0.38,0.74] | -0.7  [-3.3,0.6] | 0.62  [0.38,0.76] | 0.61  [0.33,0.76] | -3.2  [-11.4,1.2] |
|  | Quality | 3 | 0.56  [0.43,0.78] | 0.57  [0.42,0.78] | 0.7  [-1.7,4.1] | 0.56  [0.48,0.72] | 0.57  [0.47,0.74] | 1.0  [-2.4,3.4] |
|  | Seed | 5 | 0.79  [0.74,0.84] | 0.80  [0.74,0.84] | 0.3  [-0.2,0.8] | 0.92  [0.90,0.93] | 0.92  [0.90,0.94] | 0.2  [0.1,0.3] |
|  | Yield | 6 | 0.68  [0.57,0.81] | 0.68  [0.56,0.82] | 0.5  [-0.1,1.3] | 0.74  [0.66,0.82] | 0.75  [0.68,0.83] | 1.8  [0.4,3.4] |

**Supplemental Table S2.** Average [min, max] (across traits/environments-partitions) sparsity (i.e., $n_{\sup}$: the average number of training observations with a non-zero weight) of the optimal MT-SGP, average intercept and slope coefficient (SD) of the regression of observed phenotypic values on predictions, and average variance (SD) of predictions with MT-GBLUP and MT-SGP, by dataset

| **Dataset** | | **n_sup_ (%)** | **Intercept** | | **Slope** | | **Variance** | |
| --- | --- | --- | --- | --- | --- | --- | --- | --- |
|  |  | **SGP** | **GBLUP** | **SGP** | **GBLUP** | **SGP** | **GBLUP** | **SGP** |
| Wheat | | 1.30%  [0.4, 4.3] | -0.005  (0.032) | -0.010  (0.036) | 1.027  (0.080) | 1.401  (0.111) | 0.200  (0.045) | 0.132  (0.031) |
| Maize | North | 1.94%  [1.5, 2.8] | -0.001  (0.022) | 0.009  (0.024) | 1.027  (0.055) | 1.089  (0.085) | 0.540  (0.249) | 0.507  (0.248) |
|  | South | 6.30%  [1.7, 19.3] | -0.000  (0.039) | -0.001  (0.041) | 1.011  (0.106) | 1.051  (0.120) | 0.405  (0.225) | 0.384  (0.218) |
| Rice | Morphology | 2.46%  [0.5, 92.5] | 0.002  (0.087) | -0.002  (0.098) | 1.092  (0.206) | 1.379  (0.361) | 0.353  (0.185) | 0.241  (0.159) |
|  | Quality | 9.70%  [0.5, 97.7] | -0.023  (0.089) | -0.017  (0.117) | 1.030  (0.177) | 1.270  (0.236) | 0.309  (0.134) | 0.223  (0.128) |
|  | Seed | 43.68%  [1.2, 97.5] | 0.010  (0.038) | 0.009  (0.040) | 1.212  (0.071) | 1.238  (0.090) | 0.609  (0.108) | 0.592  (0.115) |
|  | Yield | 12.54%  [0.5, 98.0] | 0.012  (0.066) | 0.015  (0.073) | 1.158  (0.192) | 1.375  (0.307) | 0.432  (0.157) | 0.338  (0.152) |

3. **Supplemental Notes**

***Supplemental Note S1.* Obtaining a grid of values for the penalization parameter**

Recall the penalized prediction in which the weights $\hat{\boldsymbol{\beta}}$ are estimated by minimizing the objective function

$$\hat{\boldsymbol{\beta}}=\underset{\boldsymbol{\beta}}{arg min}\left[ \frac{1}{2}\boldsymbol{\beta}'\boldsymbol{\Sigma}\boldsymbol{\beta}-\boldsymbol{\beta}'\boldsymbol{\Gamma}+\lambda F\left( \boldsymbol{\beta} \right) \right]$$

where $\boldsymbol{\Sigma}$ and $\boldsymbol{\Gamma}$ are estimates of the (co)variance matrix of the predictors, and the covariance matrix between predictors and the response, respectively. Following Friedman et al. (2010), values for $\lambda$,

$$\lambda_{1}, \lambda_{2},\ldots,\lambda_{L}$$

can be chosen as an evenly-spaced (logarithm scale) grid of decreasing values from $\lambda_{1}=\lambda_{\max}$ to $\lambda_{L}=\lambda_{\min}$. Here, $\lambda_{\max}=\max_{i} \left\{ \frac{\left| \boldsymbol{\Gamma}_{i} \right|}{\sqrt{\boldsymbol{\Sigma}_{ii}}} \right\}$ is the smallest value of $\lambda$ that yields all weights $\hat{\boldsymbol{\beta}}$ equal to zero, and $\lambda_{\min}$ is a small number (e.g., $1\times{10}^{-5}$) that allows all weights to be non-zero. A grid of $L=100$ values of $\lambda$ can be calculated using the instructions below

```
# Sigma:(co)variance of predictors
# Gamma: variance between predictors and response
lambda.max = abs(Gamma)/sqrt(diag(Sigma))
lambda.min = 1E-5
grid = exp(seq(log(lambda.max), log(lambda.min), length=100)
```

***Supplemental Note S2***. **Using cross-validation to determine an optimal penalization value.**

A cross-validation analysis to obtain an optimal value of $\lambda$ can be implemented as in Lopez-Cruz and de los Campos (2021), as follows:

1) Partition the training data ($\mathbf{K}_{\mathrm{TRN}}$ and $\boldsymbol{y}_{\mathrm{TRN}}$) into $K$ equal-sized subsets (called *folds*). This is, we obtain $\mathbf{K}_{fold(k)}$ and $\boldsymbol{y}_{fold(k)}$ each of size $n_{\mathrm{fold}}$.
2) Obtain the weights ${\hat{\mathbf{W}}}^{\left( \lambda\right)}=({\hat{\boldsymbol{w}}}_{1},\ldots,{\hat{\boldsymbol{w}}}_{n_{\mathrm{fold}}})'$ for the $k^{\mathrm{th}}$ fold using data from the remaining $K-1$ folds for model training. This can be done using the solveEN() function by passing $\boldsymbol{\Sigma}=\sigma_{u}^{2}\mathbf{K}_{\mathrm{fold}(-k)}+\sigma_{\varepsilon}^{2}\mathbf{I}$ and $\boldsymbol{\Gamma}=\sigma_{u}^{2}\mathbf{K}_{\mathrm{fold}\left( -k \right),\mathrm{fold}(k)}$ as inputs. Here, *fold*(*-k*) indexes training data from all folds excluding the $k^{\mathrm{th}}$ fold. The weights are derived over a grid of values of the penalization parameter $\lambda_{1}, \lambda_{2},\ldots,\lambda_{L}$.
3) Derive the predictions ${\hat{\boldsymbol{g}}}_{\mathrm{fold}(k)}^{(\lambda)}={\hat{\mathbf{W}}}^{\left( \lambda\right)}\boldsymbol{y}_{\mathrm{fold}(-k)}$ for the $k^{\mathrm{th}}$ fold using the phenotypes from the remaining $K-1$ folds. We get predictions for each value of $\lambda$, i.e., ${\hat{\boldsymbol{g}}}_{\mathrm{fold}(k)}^{(\lambda_{1})}$, ${\hat{\boldsymbol{g}}}_{\mathrm{fold}(k)}^{(\lambda_{2})}$,…, ${\hat{\boldsymbol{g}}}_{\mathrm{fold}(k)}^{(\lambda_{l})}$.
4) Evaluate the within-fold prediction accuracy (PA), $\rho_{k}^{\left( \lambda\right)}=cor\left( \boldsymbol{y}_{\mathrm{fold}(k)},{\hat{\boldsymbol{g}}}_{\mathrm{fold}\left( k \right)}^{(\lambda)} \right)$ as a function of the penalization parameter $\lambda$, this is, $\rho_{k}^{\left( \lambda_{1} \right)},\rho_{k}^{(\lambda_{2})},\ldots,\rho_{k}^{(\lambda_{l})}$.
5) Obtain a unique accuracy-versus- $\lambda$ profiling $\rho^{\lambda_{1}},\rho^{\lambda_{2}},\ldots,\rho^{\lambda_{l}}$, this can be done by averaging across all folds. Choose the value $\lambda_{\mathrm{CV}}$ for which the prediction accuracy is maximum.

A 10-folds CV in a ST-SGP can be implemented using the SGP.CV() function as follows

```
# n: number of genotypes
# K: genomic relationship matrix (n x n)
# varU, varE: genetic and error variances
# y: vector of phenotypes of length n
# trn: vector indexing training set, e.g., trn=seq(1,0.8*n) first 80% of entries
fmcv = SGP.CV(y=y, K=K, trn=trn, nfolds=10, varU=varU, varE=varE)
lambda.cv = summary(fmcv)$optCOR["lambda"]    # retrieve the optimal penalization
```

4. **Supplemental Boxes**

The following boxes show the code to implement the single and multi-trait genomic prediction. All R-scripts were run on a High-Performance Computing Center (HPCC) (<https://docs.icer.msu.edu/Cluster_Resources/>). Scripts were submitted as a batch job script using the shebang line #!/usr/bin/env Rscript. Job requirements (e.g., memory, number of CPUs, run time) were specified using the SLURM scheduler by adding the prefix #SBATCH at each request instruction line in the script header.

To run the scripts for multi-job implementation we specified in the script header a job array through the SLURM option, e.g., #SBATCH --array=1-100, each value of the array is read in the R-code and assigned to a specific job with the instruction job <- Sys.getenv("SLURM_ARRAY_TASK_ID").

**2.1 Data preparation**

The data used in this study is provided in Supplemental File S1 which includes phenotypic and genotypic data. Phenotypic file (pheno.csv) consists of a matrix with grain yield records for $n=3,731$ wheat lines (in rows) evaluated at four environments (B2I, B5I, MEL, and LHT) in columns. Genotypic data (geno.csv) is a matrix with 9,045 SNP markers (in columns) for all the $3,731$ wheat lines (in rows).

The following code was used to (i) obtain a genomic relationship matrix (GRM). The second part (ii) is used to obtain training-testing partitions in which, within each environment, we randomly assigned 70% of the lines for training and used the remaining 30% of the lines for testing. In part (iii) we created folds for cross-validation within each training set for the single- and multi-environment models.

***Supplemental Box S1***

```
library(SFSI)

setwd("~/Dropbox/PFI/SFSI_paper/pipeline")

# Load source data
Y <- read.csv("data/pheno.csv", row.names=1)
X <- read.csv("data/geno.csv", row.names=1)

#=====================================
# For demonstration purposes we use a subset of the data
#=====================================
Y <- scale(Y)[1:500,]
X <- X[1:500,]

#=====================================
# Part 1. Genomic relationships matrix (GRM)
#=====================================
K <- tcrossprod(scale(X, center=TRUE, scale=FALSE))
K <- K/mean(diag(K))

# Save data
save(X, file="data/X.RData")
save(Y, file="data/Y.RData")
save(K, file="data/K.RData")

#=====================================
# Part 2. Get CV partitions: TRN,TST
#=====================================
nRep <- 100   # Number of replicates of training-testing partitions
pTST <- 0.3   # Proportion of the data to assign to testing set
ng <- 2

n <- nrow(Y)
ntraits <- ncol(Y)
nTST <- round(n*pTST)
cc <- t(combn(ntraits,ng))
nComb <- floor(nTST/(ntraits-1))
seeds <- round(seq(1E3, 1E7, length=nRep))

# TRN and TST sets (0=tst, 1=trn)
SETS <- matrix(NA,nrow=n*ntraits,ncol=nRep)
colnames(SETS) <- paste0("rep",1:nRep)

for(rr in 1:nRep){
  set.seed(seeds[rr])
  index <- sample(1:n, nComb*nrow(cc))
  sets <- matrix(1, nrow=n, ncol=ntraits)
  for(i in 1:length(index)){
    k <- ifelse((i%%nrow(cc))==0, nrow(cc), i%%nrow(cc))
    sets[index[i], cc[k,]] <- 0
  }
  SETS[,rr] <- as.vector(sets)
}

INFO_SETS <- data.frame(geno=as.vector(row(Y)), trait=colnames(Y)[col(Y)])

save(SETS, INFO_SETS, file="data/CV_trn_tst_sets.RData")

#=====================================
# Part 3. Get folds within each training set
#=====================================
nfolds <- 10
nTRN <- n-nTST

# Single trait folds
FOLDS <- get_folds(n=nTRN, k=nfolds, nCV=nRep)
save(FOLDS,  file=paste0("data/CV_",nfolds,"_folds_single_trait.RData"))

# Multi trait folds
FOLDS <- get_folds(n=nTRN*ntraits, k=nfolds, nCV=nRep)
save(FOLDS,  file=paste0("data/CV_",nfolds,"_folds_multi_trait.RData"))

#=====================================
# Part 4. Create some structure of folders
#=====================================
dir.create("output", recursive=TRUE)
dir.create("misc", recursive=TRUE)

#=====================================
# Part 5. Create a file with useful functions
#=====================================
write(
"getG0i <- function(Z, Bi){
   U <- Z%*%Bi
   G0i <- cov(U)
   return(G0i[row(G0i)>=col(G0i)])
}

getG0 <- function(X, B){
  q <- dim(B)[3]
  G <- t(apply(FUN=getG0i, X=B, Z=X, MARGIN=1))
  return(G)
}", file="misc/functions.R")
```

**2.2 Genetic and error variances estimation**

The following code was used to obtain the genetic ($\mathbf{G}_{0}$) and error ($\mathbf{R}$) (co)variance matrices for the multi-environment models, and the genetic ($\sigma_{u}^{2}$) and error ($\sigma_{\varepsilon}^{2}$) variances for the single-environment models. These tasks are performed within the training set within a given training-testing partition. The script was run for multi-job implementation, each job representing a different training-testing partition replicate.

***Supplemental Box S2***

```
setwd("~/Dropbox/PFI/SFSI_paper/pipeline")

library(SFSI)
library(BGLR)
source("misc/functions.R")

load("data/X.RData")
load("data/Y.RData")
load("data/K.RData")
load("data/CV_trn_tst_sets.RData")

JOBS <- expand.grid(replicate=1:5)
job <- as.integer(Sys.getenv("SLURM_ARRAY_TASK_ID", "1"))
replicate <- as.vector(JOBS[job,"replicate"])

# Read trn/tst sets
trn_tst <- matrix(SETS[,replicate], ncol=ncol(Y))

outdir <- "output/var_comps"
if(!file.exists(outdir)) dir.create(outdir, recursive=TRUE)

yNA <- Y[]
yNA[trn_tst==0] <- NA

#============ Multi-trait model ============#
nIter=350; burnIn=50; thin=10  # we used nIter=35000; burnIn=5000

# Genetic and residual covariances (method in Lehermeier et al., 2017)
Z <- scale(X, center=TRUE, scale=TRUE)/sqrt(ncol(X))
ETA <- list(list(X=Z, model="BRR", saveEffects=TRUE))
tmp <- paste0(outdir,"/",tempfile(tmpdir=""),"_")
fm <- Multitrait(y=yNA, ETA=ETA, resCov=list(type="DIAG"),
                 nIter=nIter, burnIn=burnIn, thin=thin, saveAt=tmp)

B <- readBinMatMultitrait(paste0(tmp,'ETA_1_beta.bin'))
B <- B[-(1:(round(burnIn/thin))),,]
G0 <- xpnd(colMeans(getG0(Z,B)))
R <- fm$resCov$R
b <- fm$mu

unlink(paste0(tmp,"*"))
save(G0,R,b, file=paste0(outdir,"/varcomps_multi_rep_",replicate,".RData"))

#============ Single-trait model ============#
fm2 <- fitBLUP(y=yNA, K=K)
varU <- fm2$varU
varE <- fm2$varE
b <- fm2$b

save(varU,varE,b, file=paste0(outdir,"/varcomps_single_rep_",replicate,".RData"))
```

**2.3 Prediction accuracy versus sparsity of the index**

The code below was used to derive multi-environment SGP predictions for the testing individuals within a given training-testing partition. This is done across 100 values of the penalization parameter ($\lambda$). The analysis is performed in 10 small chunks of one tenth of the total testing set size, specified through the argument ‘subset’, for instance, SGP(...,subset=c(1,10)). The script was run for multi-job implementation, each job representing a combination of chunk and training-testing partition replicate.

***Supplemental Box S3***

```
setwd("~/Dropbox/PFI/SFSI_paper/pipeline")

library(SFSI)

nchunks <- 10

JOBS <- expand.grid(chunk=1:nchunks, replicate=1:5)
job <- as.integer(Sys.getenv("SLURM_ARRAY_TASK_ID", "1"))
replicate <- as.vector(JOBS[job,"replicate"])
chunk <- as.vector(JOBS[job,"chunk"])

load("data/Y.RData")
load("data/K.RData")
load("data/CV_trn_tst_sets.RData")

# Read variance components
load(paste0("output/var_comps/varcomps_multi_rep_",replicate,".RData"))

# Read trn/tst sets
trn_tst <- SETS[,replicate]

outdir <- paste0("output/curve_multitrait/rep_",replicate,"/")
if(!file.exists(outdir)) dir.create(outdir, recursive=TRUE)

#============ Multi-trait model ============#
y <- as.vector(Y)
ID_geno <- as.vector(row(Y))
ID_trait <- as.vector(col(Y))
trn <- which(trn_tst==1)
tst <- which(trn_tst==0)

SGP(y=y, K=K, b=b, varU=G0, varE=R, subset=c(chunk,nchunks),
    ID_geno=ID_geno, ID_trait=ID_trait, trn=trn, tst=tst, save.at=outdir)
```

The code below was used to collect the predictions from all chunks within a given training-testing partition (obtained after running code in *Supplemental* *Box S3*). This task is implemented for all training-testing partitions replicates using a ‘for’ loop.

***Supplemental Box S4***

```
setwd("~/Dropbox/PFI/SFSI_paper/pipeline")

library(SFSI)

for(replicate in 1:5){
  path <- paste0("output/curve_multitrait/rep_",replicate,"/")
  fm <- read_SGP(path=path)
  summary(fm, save.at=path)
  unlink(paste0(path,"beta_i_*.bin"))
}
```

The code below was used to create a plot showing the prediction accuracy of the multi-environment SGP as a function of the sparsity of the index, by environment (Figure 2).

***Supplemental Box S5***

```
setwd("~/Dropbox/PFI/SFSI_paper/pipeline")

library(SFSI)

load("data/Y.RData")

paths <- paste0("output/curve_multitrait/rep_",1:5,"/")
fm <- read_summary(path=paths)

multitrait.plot(fm, trait_names=colnames(Y), main=NULL, pch=c("*",19),
                xlab=expression("Average support set size ("*n[sup]*")"),
                ylab="Prediction accuracy (PA)", point.size=c(6,1.3),
                point.color="red", legend.position=c(0.93,0.01))
```

**2.4 Optimal SGP using a cross-validated penalization parameter**

***2.4.1. Multi-environment SGP***. In this section we show the code used to perform internal 10-folds cross-validation (CV) within the training set within a given training-testing partition, for the multi-environment SGP. The analysis was performed separately for each fold (specified through the argument ‘subset’). The script was run for multi-job implementation, each job representing a combination of fold and training-testing partition replicate.

***Supplemental Box S6***

```
setwd("~/Dropbox/PFI/SFSI_paper/pipeline")

library(SFSI)

JOBS <- expand.grid(fold=1:10, replicate=1:5)
job <- as.integer(Sys.getenv("SLURM_ARRAY_TASK_ID", "1"))
replicate <- as.vector(JOBS[job,"replicate"])
fold <- as.vector(JOBS[job,"fold"])

load("data/Y.RData")
load("data/K.RData")
load("data/CV_trn_tst_sets.RData")
load("data/CV_10_folds_multi_trait.RData")

# Read variance components
load(paste0("output/var_comps/varcomps_multi_rep_",replicate,".RData"))

# Read trn/tst sets and folds within trn set
trn_tst <- SETS[,replicate]
folds <- FOLDS[,replicate,drop=F]

outdir <- paste0("output/CV_multitrait/rep_",replicate,"/")
if(!file.exists(outdir)) dir.create(outdir, recursive=TRUE)

#============ Multi-trait model ============#
y <- as.vector(Y)
ID_geno <- as.vector(row(Y))
ID_trait <- as.vector(col(Y))
trn <- which(trn_tst==1)

SGP.CV(y=y, K=K, b=b, varU=G0, varE=R, subset=fold,
       ID_geno=ID_geno, ID_trait=ID_trait, trn=trn, folds=folds, save.at=outdir)
```

The code below was used to collect results from all folds in the cross-validation (obtained after running code in *Supplemental* *Box S6*) and to obtain the optimal value of the penalization. This task is implemented for all training-testing partitions replicates using a ‘for’ loop.

***Supplemental Box S7***

```
setwd("~/Dropbox/PFI/SFSI_paper/pipeline")

library(SFSI)

for(replicate in 1:5){
  path <- paste0("output/CV_multitrait/rep_",replicate,"/")
  fm <- read_SGP(path=path, type="SGP.CV")
  summary(fm, save.at=path)
}
```

The code below was used to derive the optimal multi-environment SGP using the penalization value obtained from cross-validation (obtained after running code in *Supplemental* *Box S7*). The multi-environment GBLUP model (i.e., the multi-environment SGP with $\lambda=0$) is also derived. The script was run for multi-job implementation, each job representing a different training-testing partition replicate.

***Supplemental Box S8***

```
setwd("~/Dropbox/PFI/SFSI_paper/pipeline")

library(SFSI)

JOBS <- expand.grid(replicate=1:5)
job <- as.integer(Sys.getenv("SLURM_ARRAY_TASK_ID", "1"))
replicate <- as.vector(JOBS[job,"replicate"])

load("data/Y.RData")
load("data/K.RData")
load("data/CV_trn_tst_sets.RData")

# Read variance components
load(paste0("output/var_comps/varcomps_multi_rep_",replicate,".RData"))

# Read trn/tst sets
trn_tst <- SETS[,replicate]

# Read optimal lambda
ss <- read_summary(paste0("output/CV_multitrait/rep_",replicate,"/"), type="SGP.CV")
lambda <- as.vector(ss$optCOR["lambda"])

outdir <- "output/opt_multitrait"
if(!file.exists(outdir)) dir.create(outdir, recursive=TRUE)

#============ Multi-trait model ============#
y <- as.vector(Y)
ID_geno <- as.vector(row(Y))
ID_trait <- as.vector(col(Y))
trn <- which(trn_tst==1)
tst <- which(trn_tst==0)

# GBLUP (lambda = 0)
fm1 <- SGP(y=y, K=K, b=b, varU=G0, varE=R, lambda=0,
           ID_geno=ID_geno, ID_trait=ID_trait,
           trn=trn, tst=tst)

# SGP
fm2 <- SGP(y=y, K=K, b=b, varU=G0, varE=R, lambda=lambda,
           ID_geno=ID_geno, ID_trait=ID_trait,
           trn=trn, tst=tst)

out <- cbind(summary(fm1)$accuracy[1,-1], summary(fm2)$accuracy[1,-1])
dimnames(out) <- list(colnames(Y),c("GBLUP","SGP"))

save(out, file=paste0(outdir,"/results_rep_",replicate,".RData"))
```

***2.4.2. Single-environment SGP***. In this section we show the code used to perform internal 10-folds cross-validation within the training set within a given training-testing partition, for the single-environment SGP. The script was run for multi-job implementation, each job representing a different training-testing partition replicate.

***Supplemental Box S9***

```
setwd("~/Dropbox/PFI/SFSI_paper/pipeline")

library(SFSI)

JOBS <- expand.grid(replicate=1:5)
job <- as.integer(Sys.getenv("SLURM_ARRAY_TASK_ID", "1"))
replicate <- as.vector(JOBS[job,"replicate"])

load("data/Y.RData")
load("data/K.RData")
load("data/CV_trn_tst_sets.RData")
load("data/CV_10_folds_single_trait.RData")

# Read variance components
load(paste0("output/var_comps/varcomps_single_rep_",replicate,".RData"))

# Read trn/tst sets and folds within trn set
trn_tst <- matrix(SETS[,replicate], ncol=ncol(Y))
folds <- FOLDS[,replicate,drop=F]

outdir <- "output/CV_singletrait"
if(!file.exists(outdir)) dir.create(outdir, recursive=TRUE)

#============ Single-trait model ============#
for(j in 1:ncol(Y))      # loop over each environment
{
  trn <- which(trn_tst[,j]==1)

  fm <- SGP.CV(y=Y[,j], K=K, b=b[j], varU=varU[j], varE=varE[j],
               trn=trn, folds=folds)
  summary(fm, save.at=paste0(outdir,"/rep_",replicate,"_trait_",j,"_"))
}
```

The code below was used to derive the optimal single-environment SGP using the penalization value obtained from cross-validation (obtained after running code in *Supplemental* *Box S9*). The single-environment GBLUP model (i.e., the single-environment SGP with $\lambda=0$) is also derived. The script was run for multi-job implementation, each job representing a different training-testing partition replicate.

***Supplemental Box S10***

```
setwd("~/Dropbox/PFI/SFSI_paper/pipeline")

library(SFSI)

JOBS <- expand.grid(replicate=1:5)
job <- as.integer(Sys.getenv("SLURM_ARRAY_TASK_ID", "1"))
replicate <- as.vector(JOBS[job,"replicate"])

load("data/Y.RData")
load("data/K.RData")
load("data/CV_trn_tst_sets.RData")

# Read variance components
load(paste0("output/var_comps/varcomps_single_rep_",replicate,".RData"))

# Read trn/tst sets
trn_tst <- matrix(SETS[,replicate], ncol=ncol(Y))

outdir <- "output/opt_singletrait"
if(!file.exists(outdir)) dir.create(outdir, recursive=TRUE)

out <- matrix(NA, nrow=ncol(Y), ncol=2, dimnames=list(colnames(Y),c("GBLUP","SGP")))

#============ Single-trait model ============#
for(j in 1:ncol(Y)){      # loop over each environment
{
  trn <- which(trn_tst[,j]==1)
  tst <- which(trn_tst[,j]==0)

  # GBLUP
  fm1 <- SGP(y=Y[,j], K=K, b=b[j], varU=varU[j], varE=varE[j],
            lambda=0, trn=trn, tst=tst)

  # SGP
  # Read optimal lambda
  ss <- read_summary(paste0("output/CV_singletrait/rep_",replicate,"_trait_",j,"_"))
  lambda <- as.vector(ss$optCOR["lambda"])

  fm2 <- SGP(y=Y[,j], K=K, b=b[j], varU=varU[j], varE=varE[j],
            lambda=lambda, trn=trn, tst=tst)

  out[j,] <- c(summary(fm1)$accuracy, summary(fm2)$accuracy)
}

save(out, file=paste0(outdir,"/results_rep_",replicate,".RData"))
```

***2.4.3. Sparse versus non-sparse genomic prediction in single and multi-environment models***. The following code can be used to create a plot showing the prediction accuracy achieved by the multi-environment sparse and non-sparse genomic prediction models (obtained after running code in *Supplemental* *Box S8*). For comparison, results of the single-environment models (obtained after running code in *Supplemental* *Box S10*) are added to the plot (Figure 3).

***Supplemental Box S11***

```
setwd("~/Dropbox/PFI/SFSI_paper/pipeline")

OUT <- c()
for(rr in 1:5)
{
  #============ Single-trait model ============#
  filename <- paste0("output/opt_singletrait/results_rep_",rr,".RData")
  if(file.exists(filename)){
    load(filename, verbose=F)
    tmp <-  reshape2::melt(out, value.name="accuracy")
    OUT <- rbind(OUT, data.frame(rep=rr, Type="Single-environment",tmp))
  }

  #============ Multi-trait model ============#
  filename <- paste0("output/opt_multitrait/results_rep_",rr,".RData")
  if(file.exists(filename)){
    load(filename, verbose=F)
    tmp <-  reshape2::melt(out, value.name="accuracy")
    OUT <- rbind(OUT, data.frame(rep=rr, Type="Multi-environment",tmp))
  }
}

colnames(OUT)[colnames(OUT)=="Var1"] <- "trait"
colnames(OUT)[colnames(OUT)=="Var2"] <- "model"

dat <- OUT
models <- c("GBLUP","SGP")
dx <- 0.18

dat$model2 <- paste(dat$model, dat$Type)
dat$model <- factor(dat$model, levels=models)
dat$Type <- factor(dat$Type, levels=c("Single-environment","Multi-environment"))
tmp <- paste(rep(levels(dat$model),nlevels(dat$Type)),rep(levels(dat$Type),each=nlevels(dat$model)))
dat$model2 <- factor(dat$model2, levels=tmp)
dat$x0 <- as.numeric(dat$Type)
dat$x <- ifelse(dat$model=="GBLUP",dat$x0-dx,dat$x0+dx)

dat0 <- do.call(rbind,lapply(split(dat, paste(dat$trait,dat$Type)),function(dt){
          xx <- data.frame(do.call(rbind,lapply(split(dt,dt$rep),function(z){
            rownames(z) <- z$model
            t(z[models, "accuracy",drop=F])
          })))
          a1 <- apply(xx,2,function(z)sprintf('%.3f',mean(z)))
          a2 <- sum(xx$SGP>xx$GBLUP)/nrow(xx)
          data.frame(dt[1,c("trait","Type","model2")],t(a1),Pr=a2, x=as.numeric(dt[1,"Type"]),
                     y1=max(xx[,models[1]]),y2=max(xx[,models[2]]))
        }))

dat0 <- data.frame(dat0, y=min(dat$accuracy),
                   label=paste0("P=",sprintf('%.2f',dat0$Pr)))

color0 <- c(GBLUP="#56B4E9", SGP="#E69F00")

library(ggplot2)

ggplot(dat, aes(x, accuracy, group=model2)) +
       stat_boxplot(geom="errorbar", width=0.75*dx, position="dodge") +
       geom_boxplot(aes(group=model2, fill=model), position="dodge",
                    width=2*0.75*dx, outlier.size=0.5, outlier.shape=1) +
       facet_wrap(~trait, scales="free_y") + theme_bw() +
       labs(y="Prediction accuracy (PA)", x=NULL, fill=NULL) +
       scale_fill_manual(values=color0) +
       scale_x_continuous(breaks=c(1,2),labels=levels(dat$Type)) +
       geom_text(data=dat0, aes(x,y=-Inf,label=label), color="gray20", vjust=-0.5, size=2.6) +
       geom_text(data=dat0, aes(x=x-dx,y=y1,label=GBLUP), color="red4", vjust=-0.4, size=2.6) +
       geom_text(data=dat0, aes(x=x+dx,y=y2,label=SGP), color="red4", vjust=-0.4, size=2.6)
```

**REFERENCES**

Friedman, J., Hastie, T., & Tibshirani, R. (2010). Regularization paths for generalized linear models via coordinate descent. *Journal of Statistical Software*, 33(1), 1–22.

Lehermeier, C., de los Campos, G., Wimmer, V., & Schon, C.C. (2017). Genomic variance estimates: with or without disequilibrium covariances? *Journal of Animal Breeding & Genetics*. 134(3):232–241

Lopez-Cruz, M., & de los Campos, G. (2021). Optimal breeding-value prediction using a Sparse Selection Index. *Genetics*, *218*(1), 1–10.

Lopez-Cruz, M., Dreisigacker, S., Crespo-Herrera, L., Bentley, A. R., Singh, R., Poland, J., et al. (2022). Sparse kernel models provide optimization of training set design for genomic prediction in multiyear wheat breeding data. *Plant Genome* e20254, 1–15.
